# Supplementary material for: The Lanthipeptide Synthetase-like Protein CA_C0082 Is an Effector of Agr Quorum Sensing in Clostridium acetobutylicum
Source: Microorganisms. 2023 May 31;11(6):1460. doi: 10.3390/microorganisms11061460 (PMC10301443; doi:10.3390/microorganisms11061460)
Supplement: Supplementary file 1 [file microorganisms-11-01460-s001.zip › microorganisms-2409254-supplementary-updated.pdf]

microorganisms

MDPI

Article

The Lanthipeptide Synthetase-Like Protein CA_C0082 Is an Effector of Agr Quorum Sensing in *Clostridium acetobutylicum*

Jonathan R. Humphreys [1,†], Zak Bean [1,†], Jamie Twycross [2] and Klaus Winzer [1,*]

[1] BBSRC/EPSRC Synthetic Biology Research Centre (SBRC), School of Life Sciences, University Park, The University of Nottingham, Nottingham NG7 2RD, UK; jonathan.humphreys@nrel.gov (J.R.H.); zak.bean@chainbiotech.com (Z.B.)
[2] School of Computer Science, Jubilee Campus, The University of Nottingham, Nottingham NG8 1BB, UK; jamie.twycross@nottingham.ac.uk
* Correspondence: klaus.winzer@nottingham.ac.uk
† These authors contributed equally to this work.

Supplementary Materials

Supplementary Figures

```
C. acetobutylicum   -MNLKEQL----NKVNDKFIKGLGKASMKIGEQANGKCVLVTLYEPKMPEELLKENIDK- 54
C. felsineum        -MNLKKQV----NKIGEKFIEGIGKASMKIGEQASDTCVLITLYEPKMPEELLKENLNK- 54
C. aurantibutyricum -MNLKKQV----NKISEKLIEGIGNVSIKVGEQATDICVLATLYEPKMPEELLKENIDK- 54
C. roseum           -MNLKKQV----NKISEKLIEGIGNVSIKVGEQATDICVLTTLYEPKMPEELLKGNIDK- 54
Clostridium sp. CT7 -MSLKNQL----NKVNDKVINSIGKASMKVGNRAFGVCMVFAIYEPKIPDALIKENYKRK 55
C. beijerinckii     MKSMRKQITSTGERLSDKIINGICDVSIKIGEKSRGRCSMLGAYEPKISIDLLKEENK-- 58
C. diolis           MKSMRKQITSTGERLSDKIINGICDVSIKIGEKSRGRCSMLGAYEPKISIDLLKEENK-- 58
C. hungatei         MKNIIGQFASMREKVEDKLISSIGNTATRIGEQSRGLCLLVFDYEPKFPMELLMKNDEQ- 59
P. cellulosolvens   MRNMKEQIVSMREKIGSKILGEIGNATVKIGEQSRGFCILLFSYEPKVPKELLYEKIED- 59
C. cellulovorans    MRNMREEL----IKIGDMVIGEIGNEAVKLGDRARGLCLFLNVYEPVIPIELLDDIE--- 53
                      .:  :.     :: . .:  : . : ::*::: . * .   *** .   *:
```

Figure S1. Alignment of AgrD peptide sequences encoded upstream of clostridial CA_C0082 homologs. For each position, identical amino acids (*) as well as conserved (:) and semi-conserved substitutions (.) are displayed. Identical amino acids are given in bold, with the cysteine residues responsible for thiolactone ring formation in red. The grey box indicates the sequences predicted to form six-membered thiolactone ring structures in the respective mature AIPs. Numbers indicate the total length of the AgrD sequence (see Table S2 for individual sequences, accession numbers and locus tags).

```
CLBEIC_16210     YKNIE-FEDRDEEVLLFLIKFISITRNKEIKATIERLLTFKG---------------------KKRLNKS  555
Clocel_4251      EVCKNSLNYDVNGVVVIFSILLYIKKNAVIEHEIQQLLKIERSIGSK-----------KQC----RKKET  589
CUB90_10685      KNENIKFSIIYDSIIISIVKIYEISKDDSLLNVIQGILSTQRSLKKQ-----------IYIYDNLKWRKR  554
CA_C0082         KQMALNDVYLNDAIILTLAKLLELTGERSLVKKIKELFSIQRMKYKD-----------IF----ARWDRR  561
NL50_15050       KQMALNDVYLNDAIILTLGKLLELTGERSLVNKIKDLFSIQRMKYKD-----------IF----ARWDRR  561
CLAUR_41030      KKLELNSVFLNDSILFILAKLLETTGDERLKKYIKEILSIQKLKHSD-----------HF----GKWDIR  550
CLFE_029800      KKLELNSVFLNDSILFILVKLLEVTGDEKLKKYIKEILSIQKLKHSD-----------HF----VKWDIR  550
CLROS_13610      KKLELNSVFLNDSILFILVKLLEVTGDEKLKKYIKEILSIQKLKHSD-----------HF----VKWDIR  551
K412_RS0114320   ISTTPGYYHGINGVIIVLINLLNLIGDTAIKSVIEELLKIERNFKFD-----------KRFSNKTMLEVE  600
CLHUN_10740      ADAASGFGDGSSGIIAVLMRLLNMTGNSVVKITIEELLKMERLVITG-----------ERLQEKAGWRNG  602
Bccel_4199       ENTNFGFGYGNDGVIAVLARFMAITGNFAIENTIKELLKKERDFNTC-----------EMIFEKPSWQSG  600
LanM2            QNYYTGFAHGTSGIAAALSRFNKVFDSQSLKKIISQCLAFEKQLYIASEKNWGSK-GREQL--SVAWCHG  909
LctM             KESIASYAHGNSGIATAFVHGYKVTKNEKYLKIFHELWNLENS--SKLRRGWTDSRKVDSS-YSSQWCHG  783
                           . :   :      .     :     :

CLBEIC_16210     HFQILLNRLKLKEVGYNDK--------LVDKEINEALSYIIKNEFN--GSKSSYYNKDIVNIEILEYAAA  615
Clocel_4251      YKKVLISRSILKEYGYNDG--------LIVQELNETIKYIIEKEFG--NG-TYYN-RDIENIELVRYAAS  647
CUB90_10685      EFNKIISRVMLRKIGFKDK--------YIKREIDSTIDYIINNNFR--NS-PYYY-GSLGSLEALKYAAY  612
CA_C0082         ILITLMGRVTLKKINFQDE--------SIDREIQQISKYIINNGFG--NS-FSCC-DDMGIIEVLKHTAA  619
NL50_15050       ILITLMGRVTLKKINFQDE--------SIDREIQQISKYVINNGFG--NS-FSCC-DDMGIIEVLKHTAA  619
CLAUR_41030      MLMTLMGRTILKKINFEYE--------NIDDEIEEFTNYIIKNGFG--NS-ISYC-NEIWCIGALKYTST  608
CLFE_029800      MLMSLMGRAILKNINFEYT--------NLDEEIEEFTKYIIKNGFG--NS-ISYC-NEIWCIDALKYTAA  608
CLROS_13610      MLMSLMGRAILKNINFEYT--------NLDEEIEEFTKYIIKNGFG--NS-ISYC-NEIWCIDALKYTAA  609
K412_RS0114320   HIGILLSRVFLKQYGYDDN--------LIDIEIDKALAWTIKKGFG--NS-PFYYNGDIGALEVLEYTAK  658
CLHUN_10740      YSGILLSRLILKKSGFEDA--------LLDEEISRAVSRTINHGFG--CS-PFYCNGDIGNLEIVDYAAE  661
Bccel_4199       WPGILLSRIILKECGYNDE--------LIDTEIYKALDYTIINGFG--NS-PYYYNGDMGNLEILEYAAE  659
LanM2            AAGILLSRSILRENGVNDP--------GLHTDILNALETTVKHGLG--NN-RSFCHGDFGQLEILRGFRE  968
LctM             ASGQAIARMEWITVNKTARFLSNSELIKVKKELGELIDILKKEGM-YTDN-FCLCHGILGNLLILNTYQE  851
                      : *     .           : ::        . :           : :  :
```

Figure S2. Structural motifs important for zinc binding and active site formation in class II (LanM-type) lanthipeptide synthetase cyclase domains are not conserved in clostridial CA_C0082 homologs. Shown is a partial sequence alignment of the C-terminal cyclase domains of identified CA_C0082 homologs and selected class II lanthipeptide synthases, LctM of *L. lactis* and LanM2 of *B. licheniformis* (see Table S1 for sequences and accessions numbers). For each position, identical amino acids (*) as well as conserved (:) and semi-conserved substitutions (.) are displayed. For three structural LanM motifs, GXAHG, WCXG and CHG (1), amino acids are shown in colour: two cysteines and one histidine involved in zinc binding: red; a catalytic site histidine: green; other conserved residues within the named motifs: blue.

```
CLBEIC_16210     100  --------ARIK-EIRVFLDNETVI-VIFTDNSKVLFFKYNILKKYSLFNEVVCYLNKKI  149
Clocel_4251      97   --------IEIV-KIDSYLLDGSAI-IWFSDGKSIVFKM-RALEDIERFNYIINWTNSKL  145
CUB90_10685      50   --------LKVK-EIKYYMKEERAI-ITFTNNKKIAVQT-YVSEDEKIINRFIWWINEKV  98
CA_C0082         60   --------VAIK-EIKYYEKEERAI-VTFTDNEKVVFQS-YISKDEEIINKFIRWINQKV  108
NL50_15050       60   --------VAIK-EIKYYEKEERAI-VTFTDNEKVVFQS-YISKDEEIINKFIRWINQKV  108
CLAUR_41030      48   --------LEIR-EIKYFIKEGRAI-VTFTSNEKIVFQS-YESKDEKIINDLIEWANKRV  96
CLFE_029800      48   --------LEIR-EIKYFIKEGRAI-VTFTSNEKIVFQS-YESKDEKIINKLIEWANKKV  96
CLROS_13610      48   --------LEIR-EIKYFIKEGRAI-VTFTSNEKIVFQS-YESKDEKIINKLIEWANKKV  96
K412_RS0114320   99   --------LKIV-DIHCSLVKRSAV-ITFSHNRKIIFRN-SSLNDEQIFNSIIRWVNFKV  147
CLHUN_10740      100  --------IKIS-EIRCNLLKKSAV-ITFTNNSRIIYKD-TMVANDIIFNNIIGWLNTRV  148
Bccel_4199       97   --------IKIA-NIKCSLIKKSAV-ITFSNSAKIVFKA-DPLKNNEIFNNIIKWSNSKV  145
LanM2            226  HIAGDPVHIELGVGDSHNKGKMAAI-LTYSDGKKIVYKP-RSHDVDDAFQLLLSWINDRN  283
LctM             134  --------M----KIKGDLHNGKAVIEITTNKSKLIYKP-KSLSNDVFFNNFLKYMDSFF  180
                                          .  .:    :    :             :: .: : :

CLBEIC_16210     200  KNVN-----------------ELFSLRDSIIDNNFS-ASNIAHHMLDSSVYNIDFLPLNK  241
Clocel_4251      196  NKVEKKDIISLRNCPSILNCTNLFIVQEEIFNQEIS-STDIAKKILDYSVYNIEFLSEDM  254
CUB90_10685      152  KEIRSSNIVDVRNYPILDRIKKVFYFNNEVPNFNFSSANQIAERVIKFSVYNIEFLPKSK  211
CA_C0082         162  SKIKSKNIIDMEDCPILDEVKDAFYSTNDVPSFNFS-ANEVAEKLVKYSVYNIEFLPESK  220
NL50_15050       162  SKIKSKNIIDMEDCPILDEVKDAFYSTNDVPSFNFS-ANEVAEKLVKYSVYNIEFLPESK  220
CLAUR_41030      150  GKLKSDNIIDMEGNPILDRVRNVISSTNEVPNFNFS-ADEIAEKIVKDSVYNMEFLPESK  208
CLFE_029800      150  EKLKSDNIIDMENNPILDRIRNVISSTNEVPNFNFS-ANEIAEKIVKDSVYNIEFLPESK  208
CLROS_13610      150  EKLKSDNIIDMENNPILDRIRNVISSTNEVPNFNFS-ANEIAEKIVKDSVYNIEFLPESK  208
K412_RS0114320   199  KNFQEGKLLKLSRLPVLTNLEGLFSISERKLNFAPS-SKNIAQKIIDSSVYNLCIIPKRH  257
CLHUN_10740      201  SNFKCKRIVTLAMYPVLMNLDGIFLAHKKKSDLNIS-SKSIAQDIIDSSVYNIEFIPKQS  259
Bccel_4199       199  KDYKNISIIPQPRYPALKDVDDIFVSMNKKLNFNIS-SKSIAHDIIESSVYKIGFLPGRL  257
LanM2            336  VDFHHENIIASGEHPVLIDLESIFHQYKKRDEPGST-AVDKANYILSRSVRSTGILPFNL  394
LctM             241  TDLHFENVISQGENPCIIDLETMFNMPMFVKDYKNE-SRNIINGKIMDSVVSTGMLPVLG  299
                       . .                   :       .     : .  .  :  ** .  ::

CLBEIC_16210     244  ------------NLVNFNIGAIKSGFEYMYNITLSNKSEFISFI----------------  276
Clocel_4251      257  ------------CLAKNYINLIKSGYKYRYNIISSNKSQLIKII----------------  289
CUB90_10685      214  ------------NIDRECIYQIKHGFEYMYSLVMNNKMELINLI----------------  246
CA_C0082         223  ------------SITEEEIYHIKCGFEYIYNMIMYNKLELIEVM----------------  255
NL50_15050       223  ------------SITEEEIYHIKCGFEYIYNMIMYNKLELIEVM----------------  255
CLAUR_41030      211  ------------EVTEEEIYNIKCGFEYMYNIVMCNKLELIEFL----------------  243
CLFE_029800      211  ------------EVTEEEIYNIKCGFEYMYNIVMCNKLELIEFL----------------  243
CLROS_13610      211  ------------EVTEEEIYNIKCGFEYMYNIVMCNKLELIESL----------------  243
K412_RS0114320   260  ------------YLAKLNISSIKLGFQYIYKIITNSKMEFIDFL----------------  292
CLHUN_10740      262  ------------ASAKLHIASIKAGFRYMYNIIVHSKREFIELL----------------  294
Bccel_4199       260  ------------PLAKTYVNSIKYGFVQHYNLVINNKMEFIDLL----------------  292
LanM2            444  EAKNLPTLDHQHVPVADYLHCIIEGFSAVYRLISDHGESYLATIEHFKNC-TVRNILKPT  503
LctM             353  ------NNEKRYCKPKDYVNDIIKGFEKTYKIIVKNKEKILGFLKKESSSVTCRILFRNT  405
                                        :  *  *:   * :      . :  :
```

Figure S3. Conserved residues important for class II (LanM-type) lanthipeptide synthetase dehydration domain function are not conserved in clostridial CA_C0082 homologs. Shown is an alignment of relevant sections of the N-terminal dehydratase domains of identified CA_C0082 homologs and selected class II lanthipeptide synthetases, LctM of *L. lactis* and LanM2 of *B. licheniformis* (see Table S1 for sequences and accessions numbers). For each position, identical amino acids (*) as well as conserved (:) and semi-conserved substitutions (.) are displayed. Conserved amino acids for LanM enzymes with proposed roles based on LctM site-directed mutagenesis (2) are shown in colour with numbers indicating their position in the latter: K159 (affecting both phosphorylation and elimination steps): blue; D242 (required for phosphorylation) and D259 (required for phosphorylation and elimination): red; R399 and T405 (required for elimination but not phosphorylation): green; residues identified as being conserved in LanM enzymes, based on (2) and (3): highlighted in grey.

```
CLBEIC_16210        ------------------------------------------------------------      0
Clocel_4251         ------------------------------------------------------------      0
CUB90_10685         ------------------------------------------------------------      0
CA_C0082            ------------------------------------------------------------      0
NL50_15050          ------------------------------------------------------------      0
CLAUR_41030         ------------------------------------------------------------      0
CLFE_029800         ------------------------------------------------------------      0
CLROS_13610         ------------------------------------------------------------      0
K412_RS0114320      ------------------------------------------------------------      0
CLHUN_10740         ------------------------------------------------------------      0
Bccel_4199          ------------------------------------------------------------      0
LanM2               MNEKSAGYHERLPVAQTQSPLVNDKIKYWRSLFGDDDKWLNKAVSLLSHDPLSSIAQSSV      60
LctM                ------------------------------------------------------------      0

CLBEIC_16210        ------------------------MLSKTKP-CNDVEKIIN-----------------EI      18
Clocel_4251         ------------------------MLSNNDSAKKE---CIK-----------------SI      16
CUB90_10685         ------------------------------------------------------------      0
CA_C0082            ------------------------------------------------------------      0
NL50_15050          ------------------------------------------------------------      0
CLAUR_41030         ------------------------------------------------------------      0
CLFE_029800         ------------------------------------------------------------      0
CLROS_13610         ------------------------------------------------------------      0
K412_RS0114320      ------------------------MTSYDTE----GNTTQS-----------------EI      15
CLHUN_10740         ------------------------MASYDVSSGGETDLRNN-----------------KI      19
Bccel_4199          ------------------------MSSFRIT---EHDNIED-----------------KI      16
LanM2               SQSVGLKDSRRGPWQKMQKRIFETPFSYKDSALQDSELLFDSLLTRFASAAQDALEEQNI      120
LctM                ----------------MKKKTYQF------------EKFLKNTFDQFSIK------QNEV      26

CLBEIC_16210        LLNRYLTSITISIIKDFFK-----------NIANT-NIISN-KEEFLRDILV-HFNNYKV      64
Clocel_4251         PKEKHATHGISKMVARLYK-----------S---LPDIVKD-KKEFITYLVE-YFYENIL      60
CUB90_10685         -----------------------------------------------MELKNGNFIEEII      13
CA_C0082            -------------------------------------MLGRRKNGIAMELKSIETVGDFI      23
NL50_15050          -------------------------------------MLGRRKNGIAMELKSIETVGDFI      23
CLAUR_41030         -----------------------------------------------MELKSKDIVGELI      13
CLFE_029800         -----------------------------------------------MELKSKDIVGELI      13
CLROS_13610         -----------------------------------------------MELKSKDIVGELI      13
K412_RS0114320      MMYNYFNSEIFNVISEFVN-----------ELADIADIIED-KKAFSLSLIK-NYYKINL      62
CLHUN_10740         RLNGCPDSQMADKINEFTI-----------N---LPDMVQD-KEAFATGLIE-GYFRKPA      63
Bccel_4199          MIYGCSSKEMSKILDTFYE-----------S---LPDIVQD-KKNFLISLME-SFYKNFT      60
LanM2               ILSPPLCRQVLTHLKQTLLQIAHQTLILELNILRLEDQLKG----DTPEMRYLDFNDNFL      176
LctM                LVED--------DLNDIIMNVCGKALVLMINEKREMNLLMG----NTPEERYQYFENEYS      74

CLBEIC_16210        EESK-----INDYLNLATKAI--------------RFVEKNLFQLEKEILK-EKG-----      99
Clocel_4251         DQDK-----IVEYFMFILRAI--------------KLTEKNLFDIKSKILNTDEH-----      96
CUB90_10685         DKNN-----FRKQLAFILKIK--------------SIVKDNYLVIKNKLLTTDEK-----      49
CA_C0082            SLSG-----NSRRLTSILKVK--------------RLIRENYSIIRSNLLNTEDK-----      59
NL50_15050          SLSG-----NSRRLTSILKLK--------------RLIRENYSIIKSNLLKTEDK-----      59
CLAUR_41030         S--K-----NKNRLTSISKIK--------------SLILDNTRSIRSNLLKTERN-----      47
CLFE_029800         S--K-----NKNRLTSISKIK--------------SLILDNTRSIRSNLLKTERN-----      47
CLROS_13610         S--K-----NKNRLTSISKIK--------------SLILDNSRSIRSNLLKTERN-----      47
K412_RS0114320      PETQ-----INEYLTFVMQVV--------------NMTEKHIHDILSNILLLDEK-----      98
CLHUN_10740         GDVS-----PEDYLGFVMEVI--------------NSAAASLPEIKASVLSTKDS-----      99
Bccel_4199          NKEQ-----LEDYVKFILEVV--------------NLTNSSFEEIKNYLLNTAED-----      96
LanM2               VNPGYLRTLFNEYPV-LLRLLCTKT----------DYWVQNFSELWKRLRQDREQLQAAF      225
LctM                STGKAFEEIKDKFPVIYIDLKNSINSYLKLVSQIMKDFKKDYSLLVERKIIEEHSTIST-      133
                                                                :

CLBEIC_16210        --------ARIK-EIRVFLDNETVI-VIFTDNSKVLFFKYNILKKYSLFNEVVCYLNKKI      149
Clocel_4251         --------IEIV-KIDSYLLDGSAI-IWFSDGKSIVFKM-RALEDIERFNYIINWTNSKL      145
CUB90_10685         --------LKVK-EIKYYMKEERAI-ITFTNNKKIAVQT-YVSEDEKIINRFIWWINEKV      98
CA_C0082            --------VAIK-EIKYYEKEERAI-VTFTDNEKVVFQS-YISKDEEIINKFIRWINQKV      108
NL50_15050          --------VAIK-EIKYYEKEERAI-VTFTDNEKVVFQS-YISKDEEIINKFIRWINQKV      108
CLAUR_41030         --------LEIR-EIKYFIKEGRAI-VTFTSNEKIVFQS-YESKDEKIINDLIEWANKRV      96
CLFE_029800         --------LEIR-EIKYFIKEGRAI-VTFTSNEKIVFQS-YESKDEKIINKLIEWANKKV      96
CLROS_13610         --------LEIR-EIKYFIKEGRAI-VTFTSNEKIVFQS-YESKDEKIINKLIEWANKKV      96
K412_RS0114320      --------LKIV-DIHCSLVKRSAV-ITFSHNRKIIFRN-SSLNDEQIFNSIIRWVNFKV      147
CLHUN_10740         --------IKIS-EIRCNLLKKSAV-ITFTNNSRIIYKD-TMVANDIIFNNIIGWLNTRV      148
Bccel_4199          --------IKIA-NIKCSLIKKSAV-ITFSNSAKIVFKA-DPLKNNEIFNNIIKWSNSKV      145
LanM2               HIAGDPVHIELGVGDSHNKGKMAAI-LTYSDGKKIVYKP-RSHDVDDAFQLLLSWINDRN      283
LctM                --------M----KIKGDLHNGKAVIEITTNKSKLIYKP-KSLSNDVFFNNFLKYMDSFF      180
                                        .  .:    :    :             :: .: : :

CLBEIC_16210        TK-------ENNIQSRKILSSRKYCLLDASE---LVKEKDLFKYYFKSGELLVILYLLCC      199
Clocel_4251         PD-------KYKLSTSCLLNYKRYGFIENIAN---EIPQDLEEYYFNSGQLLALLYMMDC      195
CUB90_10685         DE-------KHALYVKKAVYSKDYSFCELVESMDCKNENELFDYYFKSGELLLVLYILKC      151
CA_C0082            DK-------EHSLYVKKILYAEGCSFSEYIKPINSIGKREIFDYYFKSGELLLILYVLRC      161
NL50_15050          DK-------EHSLYVKKILYAEGCSFSEYIKPINSIGKREIFDYYFKSGELLLILYVLRC      161
CLAUR_41030         DK-------EYSLYVKKIMYSKVYSFSEYVKPVDCKNKSELFDYYFKSGELLLILYILGC      149
CLFE_029800         DK-------EYSLYVKKIMYSKGCSFSEYVKPVDCKNKNELFDYYFKSGELLLILYILGC      149
CLROS_13610         DK-------EYSLYVKKIMYSKGCSFSEYVKPVDCKNKNELFDYYFKSGELLLILYILGC      149
K412_RS0114320      DE-------RYQLYIRKIISYGSYGFLEYISS-EC-DKQDITNSFFCIGQLLSLIYILNC      198
CLHUN_10740         GS-------EYSLYTRKLICCGSHGFLEYIQSSQCG-EEELAKRFFNTGELLALLYILNC      200
Bccel_4199          SA-------DHHLNIRKMISRDTHSFIDYMQTFEYKDEKDLANYFLHSGQLLALLYILNC      198
LanM2               SG--------SPLKTLRLINKKRYGWSEFIPHETCHTKKELEGYYTRLGKLLAVLYSIDA      335
LctM                IKEGKSTKYKENFYLVNTLDMKTYGWVEYVDKKPINSFEEARNYYRKIGVLLSVAYTLNL      240
                                :     :        :           :    :   * ** : * :
```

Figure continued on next page

```
CLBEIC_16210        KNVN-----------------ELFSLRDSIIDNNFS-ASNIAHHMLDSSVYNIDFLPLNK    241
Clocel_4251         NKVEKKDIISLRNCPSILNCTNLFIVQEEIFNQEIS-STDIAKKILDYSVYNIEFLSEDM    254
CUB90_10685         KEIRSSNIVDVRNYPILDRIKKVFYFNNEVPNFNFSSANQIAERVIKFSVYNIEFLPKSK    211
CA_C0082            SKIKSKNIIDMEDCPILDEVKDAFYSTNDVPSFNFS-ANEVAEKLVKYSVYNIEFLPESK    220
NL50_15050          SKIKSKNIIDMEDCPILDEVKDAFYSTNDVPSFNFS-ANEVAEKLVKYSVYNIEFLPESK    220
CLAUR_41030         GKLKSDNIIDMEGNPILDRVRNVISSTNEVPNFNFS-ADEIAEKIVKDSVYNMEFLPESK    208
CLFE_029800         EKLKSDNIIDMENNPILDRIRNVISSTNEVPNFNFS-ANEIAEKIVKDSVYNIEFLPESK    208
CLROS_13610         EKLKSDNIIDMENNPILDRIRNVISSTNEVPNFNFS-ANEIAEKIVKDSVYNIEFLPESK    208
K412_RS0114320      KNFQEGKLLKLSRLPVLTNLEGLFSISERKLNFAPS-SKNIAQKIIDSSVYNLCIIPKRH    257
CLHUN_10740         SNFKCKRIVTLAMYPVLMNLDGIFLAHKKKSDLNIS-SKSIAQDIIDSSVYNIEFIPKQS    259
Bccel_4199          KDYKNISIIPQPRYPALKDVDDIFVSMNKKLNFNIS-SKSIAHDIIESSVYKIGFLPGRL    257
LanM2               VDFHHENIIASGEHPVLIDLESIFHQYKKRDEPGST-AVDKANYILSRSVRSTGILPFNL    394
LctM                TDLHFENVISQGENPCIIDLETMFNMPMFVKDYKNE-SRNIINGKIMDSVVSTGMLPVLG    299
                     . .                   :       .     : .  .  :  ** .  ::

CLBEIC_16210        V-----------------------------------------------------------    242
Clocel_4251         S-----------------------------------------------------------    255
CUB90_10685         S-----------------------------------------------------------    212
CA_C0082            K-----------------------------------------------------------    221
NL50_15050          K-----------------------------------------------------------    221
CLAUR_41030         K-----------------------------------------------------------    209
CLFE_029800         K-----------------------------------------------------------    209
CLROS_13610         K-----------------------------------------------------------    209
K412_RS0114320      R-----------------------------------------------------------    258
CLHUN_10740         K-----------------------------------------------------------    260
Bccel_4199          E-----------------------------------------------------------    258
LanM2               YFGRKNRDKVVDISG-MGGQEA-----------QESPFQALQIKGFFRDDIRLEHDRFEI    442
LctM                IDSLFGGD----PSGILGGTFSKEERVIINPFRDDIKFQKIVVRSVFKDHIPFFNN----    351

CLBEIC_16210        -------------NLVNFNIGAIKSGFEYMYNITLSNKSEFISFI---------------    274
Clocel_4251         -------------CLAKNYINLIKSGYKYRYNIISSNKSQLIKII---------------    287
CUB90_10685         -------------NIDRECIYQIKHGFEYMYSLVMNNKMELINLI---------------    244
CA_C0082            -------------SITEEEIYHIKCGFEYIYNMIMYNKLELIEVM---------------    253
NL50_15050          -------------SITEEEIYHIKCGFEYIYNMIMYNKLELIEVM---------------    253
CLAUR_41030         -------------EVTEEEIYNIKCGFEYMYNIVMCNKLELIEFL---------------    241
CLFE_029800         -------------EVTEEEIYNIKCGFEYMYNIVMCNKLELIEFL---------------    241
CLROS_13610         -------------EVTEEEIYNIKCGFEYMYNIVMCNKLELIESL---------------    241
K412_RS0114320      -------------YLAKLNISSIKLGFQYIYKIITNSKMEFIDFL---------------    290
CLHUN_10740         -------------ASAKLHIASIKAGFRYMYNIIVHSKREFIELL---------------    292
Bccel_4199          -------------PLAKTYVNSIKYGFVQHYNLVINNKMEFIDLL---------------    290
LanM2               GEAKNLPTLDHQHVPVADYLHCIIEGFSAVYRLISDHGESYLATIEHFKNC-TVRNILKP    501
LctM                -------NNEKRYCKPKDYVNDIIKGFEKTYKIIVKNKEKILGFLKKESSSVTCRILFRN    404
                                       :  *  *:   * :      . :  :

CLBEIC_16210        ------------------------------------------------KDIFASN-LEYL    285
Clocel_4251         ------------------------------------------------NELFKGD-HLIL    298
CUB90_10685         ------------------------------------------------KKLFNKN-ILQL    255
CA_C0082            ------------------------------------------------KVIFKED-ILKL    264
NL50_15050          ------------------------------------------------KVIFKED-ILKL    264
CLAUR_41030         ------------------------------------------------KPLLSNK-LLQL    252
CLFE_029800         ------------------------------------------------KPLLSNN-ILQL    252
CLROS_13610         ------------------------------------------------KPLLSNN-ILQL    252
K412_RS0114320      ------------------------------------------------NELFDKD-SPYL    301
CLHUN_10740         ------------------------------------------------NSQFEKDSSSYL    304
Bccel_4199          ------------------------------------------------RFLLN-SCSSCL    301
LanM2               TAHYASLLNKSYHPDFLRDAVDREVFLCRVEKFEDA-DTDIAAAKTELKELIRGD-IPYF    559
LctM                TMEYSVLLNAAKSPVYSNKREE---IFEKLSTFNRGLGNDI--IKSEIS-QINTLSIPYF    458
                                                                   :       :

CLBEIC_16210        GNILKKINILNEEDLKMQLYLIDAKFLTNESSIQQIILLGDEG-----------------    328
Clocel_4251         SHMIPKIYNFTENDLKQQLYFLDVRFVQFKYQDSNFKFLVGDT-----------------    341
CUB90_10685         SNIITSIYGMNNEDLRRQLYFIDIRFIGKTIPKTYVKFSSDKN-----------------    298
CA_C0082            SAVITNIYGLNEEDLKRQLYFIDIRFIGVKIARRKVNFCISER-----------------    307
NL50_15050          SAVITNIYGLNEEDLKRQLYFIDIRFIGVKIARRKVNFCISEK-----------------    307
CLAUR_41030         SAIITNVYGLNKEDLKRQLYFLDIRFSGVKISKTKINFSFSDK-----------------    295
CLFE_029800         STIITNVYGLNKEDLKRQLYFLDIRFSGVKISKTKINFSFSDK-----------------    295
CLROS_13610         STIITNVYGLNKEDLKRQLYFLDIRFSGVKISKTKINFSFSDK-----------------    295
K412_RS0114320      AAVSTNVYNLNMNDLNRQLYFLDYQFNIKQNYKSTTKFSDNRA-----------------    344
CLHUN_10740         PTIITKIYGLNEGDLRRQLHFLDVRFDAGRLNRTRVVFSGNSA-----------------    347
Bccel_4199          AIITTRVYGLNEGDLKRQLYFLDVRFPKKQIIKTPISFSEDGS-----------------    344
LanM2               LSKPSDTYLLNGEEEPIAAYFETPSFTRVIK--KISSFSDQDLKEQANVIRMSILAAYNA    617
LctM                NCQVDSNLIKNMDGETIFEHTLTP-FK--CFLSKYRRLCVDDMEQQVKLIRFSIQSQEQL    515
                              .        :     *           :

CLBEIC_16210        --------------PCRVDRKRFIKLAGKLGDHLIKKSIIGYSNGNISRTWINFSEMKRG    374
Clocel_4251         --------------YDNLNKEELKEIAIKLGDYIIQKSIIGVENSLTTRSWITDVKLGN-    386
CUB90_10685         --------------ENSINESELLSLANDFGEHMIQKGIIGVKDFTTSRTWINTVKKDKS    344
CA_C0082            --------------KETIDKNYFISIANDLGEHMIKRGIIGVKDFVTSRTWISTTRDGNN    353
NL50_15050          --------------KETIDKNYFISIANDLGEHMIKRGIIGVKDFVTSRTWISTTRDGNN    353
CLAUR_41030         --------------KETIDRSYCISMANDFGEHMIKRGIIGVKDFVTSRTWISTVSDEKS    341
CLFE_029800         --------------KETIDRSYCISMANDFGEHMIKRGIIGVKDFVTSRTWISTISDEKN    341
CLROS_13610         --------------KETIDRSYCISMANDFGEHMIKRGIIGVKDFVTSRTWISTISDEKN    341
K412_RS0114320      --------------PNKINLNYYLELAVKLGNNIIQNSIIGVINNNTSRTWINTVECGD-    389
CLHUN_10740         --------------VQETDREHLLDIAVRLGDYMIQKSIIGFNNFSTSRTWINTVRYGK-    392
Bccel_4199          --------------FNKIEKHRLLSLSARLGDHIIQKSIIGFNGSTISRNWITTVKADK-    389
LanM2               RHEKDAIDIDQNHPSPRSGALQPLAIAEKAADDLAEKRIEGND--GKDVTWISTVIEGVE    675
LctM                FKDGEQFSLYK---KQKGSQEDLLIAINELSSILENNAYIGTS--DDTINWMSLGIADND    570
                                              .. : :.   *        .*:.
```

Figure continued on next page

```
CLBEIC_16210      -KKSEVSDSLYDLYNGNSGMALFFVYLGEVTQKKYFINA----SLEIMQESIEVINNMSG    429
Clocel_4251       --KLELSHKCNSISYGTCGIAIFLLYLGVVTQKAYFIAA----SIESMRKVIYAMNKK--    438
CUB90_10685       -EKYLLRPLRNNLMNGSGGVALFFTYLGVVTKKDYFISI----AIEAIQGILEHINKLCI    399
CA_C0082          -QGYSLSPLSSDLMDGSSGVALFFAYLGLVTGKDYYKAI----AIEAIQDSINHINNLNN    408
NL50_15050        -QGYSLSPLSSDLMDGSSGVALFFAYLGLVTGKDYYKAI----AIEAIQDSINHINNLNN    408
CLAUR_41030       -ESYSLSPLGYSLMDGSSGIALFFAYLGCVTGKEYFKTV----AIESIQNSINHINNLNN    396
CLFE_029800       -QCYSLSPLGCSFMDGSSGVALFFAYLGLVTGKEYFKTV----AIESIQNSVNHINNLNN    396
CLROS_13610       -QCYSLSPLGCSFMDGSSGVALFFAYLGLVTGKEYFKTV----AIESIQNSVNHINNLNN    396
K412_RS0114320    --KIIISPECKNLYEGNSGIALFLLNLGVTTKKDYFINT----AIEAMRESISNISTLFR    443
CLHUN_10740       --RLIVSPKCYNLYEGNSGIALFFLYLGELTKKEYFINT----AIEAMREPVSHIDRLDG    446
Bccel_4199        --KIALSPGCYDLYEGNSGIALFLLYLGAVTKKDYFINT----AMEAMGESITFIRNLNK    443
LanM2             EISWTISPVSLDLYNGNAGIGLFMSYLSRFAKRPETYSHITEQCVFAIQRALNELKEKEE    735
LctM              --QILFESLENDIYKGISGIGLALLEYYEFYPNINT-----KKILKLIYKNIS-KDFINT    622
                       .     .:  *  *:.: :        .           :  :   :

CLBEIC_16210      --------DYQHIVYELFTLSKIYSITKSKTIEFAINKGILFIYKI--IKEGNIDNIS--    477
Clocel_4251       ---SIENHTLRESAEVTYTLLKIYMITKDEFIKSSVIKISSLAFSI--LSNSTDKLELED    493
CUB90_10685       -NDKINIDAFEGISGEIYAMYKLYEVTHDEHIRNVIEKGIEILSDV--IQKNNNKNTITG    456
CA_C0082          -NDDINIGAFKGISGEIYAMWKIYSVTRSNYLEASIENGIRALYIL--VQKSKDIDITNG    465
NL50_15050        -NDDINIGAFKGISGEIYAMWKIYSVTRSNYLEAAIENGIKALYIL--VQKSKDIDITNG    465
CLAUR_41030       -NDNVNIGAYRGIAGEIYVIWNIYKITHNMHLEDVIENGIQSLYIL--VQKSKEVDISNG    453
CLFE_029800       -NNNVNIGAYRGIAGEIYVIWNIYKITHNMHLEDAVENGIKSLYIL--VQKSKEIDISNG    453
CLROS_13610       NNNNVNIGAYRGIAGEIYVIWNIYKITHNMHLEDAVENGIKSLYIL--VQKSKEIDISNG    454
K412_RS0114320    -CSSIELGAFNGICGEIYTLSKIYSITKDENVKKAVKLGLLYVKSA--IVNQKDISLFSG    500
CLHUN_10740       --NTLIIGAFRGISGELYALSKIYSVTRDDSIKTAIRKGLNYIGTE--IDKQNRLGFFDG    502
Bccel_4199        -NNSIEIGAFRGISGELYTLSKIYSITKDKAIKEIVKEGLLLIQTF--IEKQVNTSLFNG    500
LanM2             FLKYADSGAFTGVSGYLYFLQHAGTVQKKNEWIELIHEALPVLEAV--IEQDENCDIISG    793
LctM              NNEPQNYGFYVGLIGEYSFLRKYEKVFHKTSSCNILKNILKDFTPEKCQTILPSDDVIAG    682
                                     : :   : :.      :

CLBEIC_16210      -ASYVAIILSIYDVIEC-NKTKKIIIELGNILYKNIE-----------------------    512
Clocel_4251       MEAFTIILLAIYKEKL-SDFNINRVFKIADLSYNKVLG------------FWKNEVCKNS    540
CUB90_10685       MCDVINVLALIYKSKGLS-RINGDILNLIKICYEKVIN------------DIKNENIKFS    503
CA_C0082          LCGVSCVLVSIYKDKD-SNKFNDIIMNLIRICMEKITG------------NMSSKQMALN    512
NL50_15050        LCGVLCVLVSIYKDKD-SNKFNDIIMNLIRICMEKITG------------NMSSKQMALN    512
CLAUR_41030       ICGVLGVLINIYKDKCISDKLHNMVLNLINLCSELVIK------------RMNYKKLELN    501
CLFE_029800       ICGALGVLIRIYKDNYISDKLHNMVLNLINLCRELVIK------------RINSKKLELN    501
CLROS_13610       ICGALGVLIRIYKDNYISDKLHNMVLNLINLCRELVIK------------RINSKKLELN    502
K412_RS0114320    LSGVLAVILAIYKNKHFSD-IKVNLLAIAELAYKNILV------------NMDTISTTPG    547
CLHUN_10740       LSGILAVLLSICENEAFRD-IRAEVLSSADLAYRKIAA------------NIGLADAASG    549
Bccel_4199        TSGILAVLISIYENKDFSD-IKNKVIATANMVYKRISS------------QVSCENTNFG    547
LanM2             SAGALMVLMSLYEQLDDP-----VFLKLAEKCAGHLLQHKTNIENGAAWKDPHTQNYYTG    848
LctM              EAGIIIYISNLNNYLEYRDEIDILLKS--------LSN------------KIKLKESIAS    722
                         :  : .           .          :

CLBEIC_16210      FEDRDEEVLLFLIKFISITRNKEIKATIERLLTFKG------------------KKRLNK    554
Clocel_4251       LNYDVNGVVVIFSILLYIKKNAVIEHEIQQLLKIERSI--------GSKKQC----RKKE    588
CUB90_10685       I--IYDSIIISIVKIYEISKDDSLLNVIQGILSTQRSL--------KKQIYIYDNLKWRK    553
CA_C0082          DVYLNDAIILTLAKLLELTGERSLVKKIKELFSIQRMK--------YKDIF----ARWDR    560
NL50_15050        DVYLNDAIILTLGKLLELTGERSLVNKIKDLFSIQRMK--------YKDIF----ARWDR    560
CLAUR_41030       SVFLNDSILFILAKLLETTGDERLKKYIKEILSIQKLK--------HSDHF----GKWDI    549
CLFE_029800       SVFLNDSILFILVKLLEVTGDEKLKKYIKEILSIQKLK--------HSDHF----VKWDI    549
CLROS_13610       SVFLNDSILFILVKLLEVTGDEKLKKYIKEILSIQKLK--------HSDHF----VKWDI    550
K412_RS0114320    YYHGINGVIIVLINLLNLIGDTAIKSVIEELLKIERNF--------KFDKRFSNKTMLEV    599
CLHUN_10740       FGDGSSGIIAVLMRLLNMTGNSVVKITIEELLKMERLV--------ITGERLQEKAGWRN    601
Bccel_4199        FGYGNDGVIAVLARFMAITGNFAIENTIKELLKKERDF--------NTCEMIFEKPSWQS    599
LanM2             FAHGTSGIAAALSRFNKVFDSQSLKKIISQCLAFEKQLYIASEKNWGSKGREQLSVAWCH    908
LctM              YAHGNSGIATAFVHGYKVTKNEKYLKIFHELWNLENSSKLRRGWTDSRKVDSSYSSQWCH    782
                       . :   :        .      :      :

CLBEIC_16210      SHFQILLNRLKLKEVGYNDK--------LVDKEINEALSYIIKNEFNGSKSSYYNKDIVN    606
Clocel_4251       TYKKVLISRSILKEYGYNDG--------LIVQELNETIKYIIEKEFGNG-TYYN-RDIEN    638
CUB90_10685       REFNKIISRVMLRKIGFKDK--------YIKREIDSTIDYIINNNFRNS-PYYY-GSLGS    603
CA_C0082          RILITLMGRVTLKKINFQDE--------SIDREIQQISKYIINNGFGNS-FSCC-DDMGI    610
NL50_15050        RILITLMGRVTLKKINFQDE--------SIDREIQQISKYVINNGFGNS-FSCC-DDMGI    610
CLAUR_41030       RMLMTLMGRTILKKINFEYE--------NIDDEIEEFTNYIIKNGFGNS-ISYC-NEIWC    599
CLFE_029800       RMLMSLMGRAILKNINFEYT--------NLDEEIEEFTKYIIKNGFGNS-ISYC-NEIWC    599
CLROS_13610       RMLMSLMGRAILKNINFEYT--------NLDEEIEEFTKYIIKNGFGNS-ISYC-NEIWC    600
K412_RS0114320    EHIGILLSRVFLKQYGYDDN--------LIDIEIDKALAWTIKKGFGNS-PFYYNGDIGA    650
CLHUN_10740       GYSGILLSRLILKKSGFEDA--------LLDEEISRAVSRTINHGFGCS-PFYCNGDIGN    652
Bccel_4199        GWPGILLSRIILKECGYNDE--------LIDTEIYKALDYTIINGFGNS-PYYYNGDMGN    650
LanM2             GAAGILLSRSILRENGVNDP--------GLHTDILNALETTVKHGLGNN-RSFCHGDFGQ    959
LctM              GASGQAIARMEWITVNKTARFLSNSELIKVKKELGELIDILKKEGMYTDNFCLCHGILGN    842
                        : *      .             :  ::         . :  .        :

CLBEIC_16210      IEILEYAAEVLENESL--KNRCINTYNNIVNKIIEPAIYEEMCYGNKPISLMRGIAGYGY    664
Clocel_4251       IELVRYAASSLEDDVL--INSCNSNLNRLVKEDLIENIKKQIKFEDRPLSFKYGTIGQGY    696
CUB90_10685       LEALKYAAYTLNDEKL--KNRCVNTFNEMFRVTIEPIIRKEINFGNENISLIDGVVGLAY    661
CA_C0082          IEVLKHTAAILSDEKL--NSSCIKTFNELVKKKIKPTINKEITYANENISLMNGVVGLAY    668
NL50_15050        IEVLKHTAAILSDEKL--NSSCIKTFNELVKKKIKPTINKEITYANENISLMNGVVGLAY    668
CLAUR_41030       IGALKYTSTILKDEKL--KNSCIKTFNDFVWKEIRPLINKEINYANENIALLNGVVGLAY    657
CLFE_029800       IDALKYTAAILKDEKL--ENSCIKTFNDFVWKEIRPLINKEITYANENIALLNGVVGLAY    657
CLROS_13610       IDALKYTAAILKDEKI--ENSCIKTFNDFVWKEIRPLINKEITYANENIALLNGVVGLAY    658
K412_RS0114320    LEVLEYTAKVLKDEVL--RNRCNNTFANLVENTIGPNTNDENKLWSLSMSLMKGISGYGH    708
CLHUN_10740       LEIVDYAAELLRDTGL--KERCNNTFITLVKEIIEPAVNAEAKTWNKSMSLLKGISGYGY    710
Bccel_4199        LEILEYAAEVLNNAEL--KNRCTNTFSELFAEVIEPYINHEKDYGNKPLSLMKGISGCGY    708
LanM2             LEILRGFREEFSE--L--NTIIQNTEDRLLTYFQENPFSKGVSRGVDSAGLMLGLSGVGY    1015
LctM              LLILNTYQENFDNKNINLKNEILNNYYSVCNYGLNKGWICGLGTEFYSYGLMTGISGILY    902
                  :  :      : :  :       ..   .                    .:  *  *  :
```

Figure continued on next page

```
CLBEIC_16210        SLIRMCGDRRTIPILSVGGIVYMNTGKN---------    692
Clocel_4251         RLIRIYNEDIVPQILFI-------------------     713
CUB90_10685         SLIRIEHEEIVPEILTLE------------------     679
CA_C0082            SLIRISSEKFVPKILWLE------------------     686
NL50_15050          SLIRISSEKFVPKILWLE------------------     686
CLAUR_41030         SLIKMSNEEYIPSILWID------------------     675
CLFE_029800         SLIRMSSEEYVPNILWLN------------------     675
CLROS_13610         SLIRMSSEEYVPNILWLN------------------     676
K412_RS0114320      MLLRKCSEN-IPNIVLLE------------------     725
CLHUN_10740         ILLRRYRNNSVPQILLLE------------------     728
Bccel_4199          MLLRKCS-SIVPQILWLQ------------------     725
LanM2               GMLQCQYGEELPELLQLSPPQALIKKNSKAFKRENVF    1052
LctM                GLIRQVKQKNNFGVLM--PYVD--------------     922
                     :::         ::
```

Figure S4. Alignment of the complete sequences of CA_C0082 homologs and selected experimentally confirmed class II lanthipeptide synthetases LctM and LanM2. For each position, identical amino acids (*) as well as conserved (:) and semi-conserved substitutions (.) are displayed. Amino acid color code: ed: small/hydrophobic (including aromatic); blue: acidic; magenta; basic; green: hydroxyl/sulfhydryl/amine and glycine). The alignment was carried out using ClustalOmega (https://www.ebi.ac.uk/Tools/msa/clustalo/) with default settings. See Table S1 for sequences and accessions numbers.

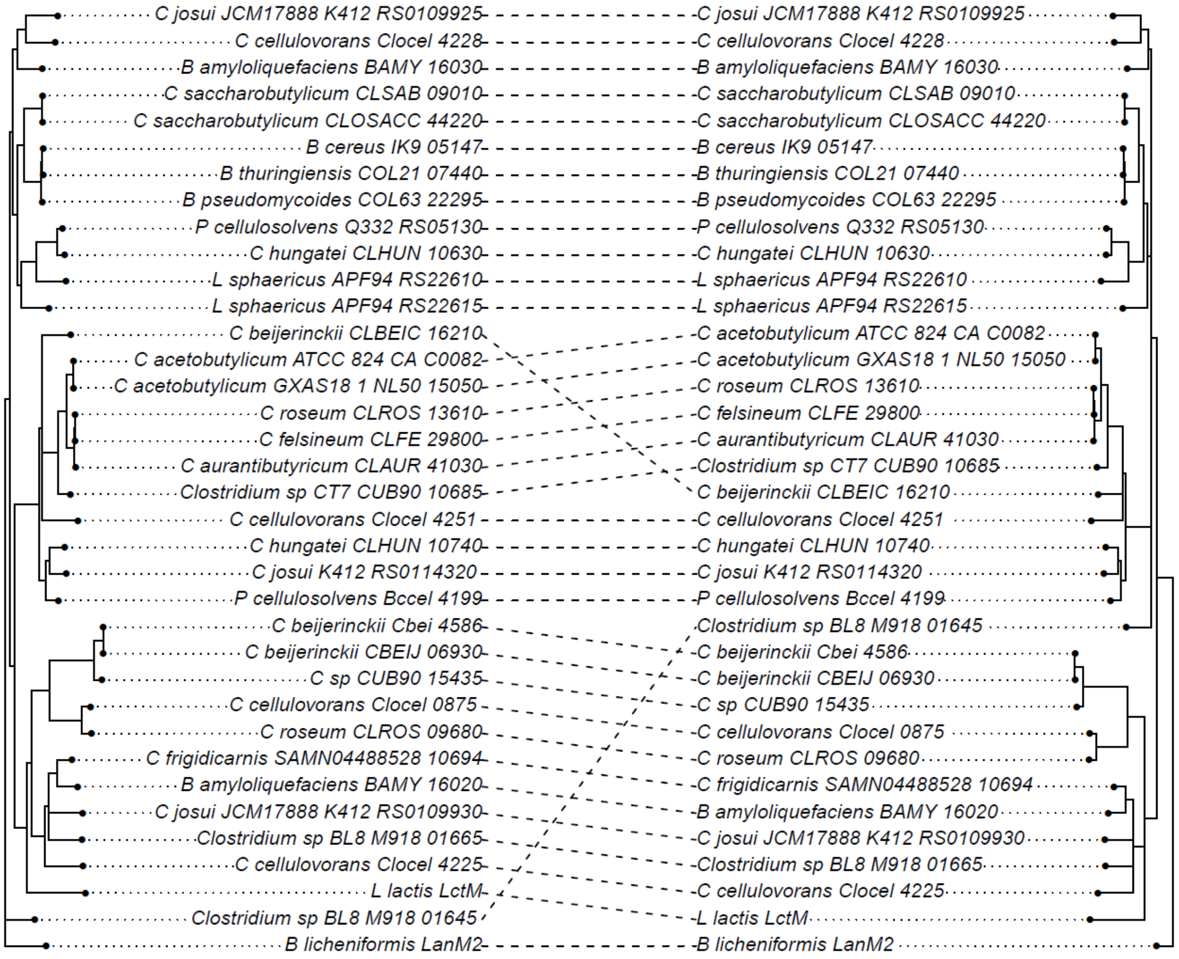

Figure S5. Comparison of phylogenetic trees generated for CA_C0082 and LanM homologs using Bayesian MCMC (left) and maximum-likelihood (right) inferences. Phylogenetic analyses were undertaken as described in the Methods using MrBayes v3.2.5 and RaxML v8.2.10. The phylogenetic analysis of CA_C0082 homologs included full length LanM proteins present in the same species, LanM homologs to which CA_C0082 homologs were most similar, and established LanM proteins, with LanM2 from *B. licheniformis* serving as an outgroup (see Table S1 for sequences used).

References for Supplementary Figures S2 and S3:

1. Paul, M., Patton, G. C. & van der Donk, W. A. Mutants of the zinc ligands of lacticin 481 synthetase retain dehydration activity but have impaired cyclization activity. *Biochemistry* **46**, 6268-6276 (2007)

2. You, Y. O., and van der Donk, W. A. Mechanistic investigations of the dehydration reaction of lacticin 481 synthetase using site-directed mutagenesis. *Biochemistry* **46,** 5991-6000 (2007)

3. Ma, H., Gao, Y., Zhao, F., Wang, J., Teng, K, Zhang, J., Zhong, J. Dissecting the catalytic and substrate binding activity of a class II lanthipeptide synthetase BovM. *Biochem. Biophys. Res. Commun.* **450**, 1126-1132 (2014)

Supplementary Tables

Table S1. List of CA_C0082 and full length LanM homologs used for Figure 1. Provided are NCBI and/or GenBank entry number(s) and locus tag/gene name.

CA_0082 homologs

Clostridium acetobutylicum ATCC 824

AE007521_1, new NCBI accession number WP_010963409.1; locus tag CA_C0082 (identical or almost identical sequences exist in strains DSM1731, DSM1732, EA2018, NCCB24020, WA and others)

MLGRRKNGIAMELKSIETVGDFISLSGNSRRLTSILKVKRLIRENYSIIRSNLLNTEDKVAIKEIKYYEKEERAIVTFTDNEKVVFQSYISKDEEIINKFIRWINQKVD

KEHSLYVKKILYAEGCSFSEYIKPINSIGKREIFDYYFKSGELLLILYVLRCSKIKSKNIIDMEDCPILDEVKDAFYSTNDVPSFNFSANEVAEKLVKYSVYNIEFLPE

SKKSITEEEIYHIKCGFEYIYNMIMYNKLELIEVMKVIFKEDILKLSAVITNIYGLNEEDLKRQLYFIDIRFIGVKIARRKVNFCISERKETIDKNYFISIANDLGEHM

IKRGIIGVKDFVTSRTWISTTRDGNNQGYSLSPLSSDLMDGSSGVALFFAYLGLVTGKDYYKAIAIEAIQDSINHINNLNNNDDINIGAFKGISGEIYAMWKIYSVTRS

NYLEASIENGIRALYILVQKSKDIDITNGLCGVSCVLVSIYKDKDSNKFNDIIMNLIRICMEKITGNMSSKQMALNDVYLNDAIILTLAKLLELTGERSLVKKIKELFS

IQRMKYKDIFARWDRRILITLMGRVTLKKINFQDESIDREIQQISKYIINNGFGNSFSCCDDMGIIEVLKHTAAILSDEKLNSSCIKTFNELVKKKIKPTINKEITYAN

ENISLMNGVVGLAYSLIRISSEKFVPKILWLE

Clostridium acetobutylicum GXAS18-1

KHD35037.1; NL50_15050

MLGRRKNGIAMELKSIETVGDFISLSGNSRRLTSILKLKRLIRENYSIIKSNLLKTEDKVAIKEIKYYEKEERAIVTFTDNEKVVFQSYISKDEEIINKFIRWINQKVD

KEHSLYVKKILYAEGCSFSEYIKPINSIGKREIFDYYFKSGELLLILYVLRCSKIKSKNIIDMEDCPILDEVKDAFYSTNDVPSFNFSANEVAEKLVKYSVYNIEFLPE

SKKSITEEEIYHIKCGFEYIYNMIMYNKLELIEVMKVIFKEDILKLSAVITNIYGLNEEDLKRQLYFIDIRFIGVKIARRKVNFCISEKKETIDKNYFISIANDLGEHM

IKRGIIGVKDFVTSRTWISTTRDGNNQGYSLSPLSSDLMDGSSGVALFFAYLGLVTGKDYYKAIAIEAIQDSINHINNLNNNDDINIGAFKGISGEIYAMWKIYSVTRS

NYLEAAIENGIKALYILVQKSKDIDITNGLCGVLCVLVSIYKDKDSNKFNDIIMNLIRICMEKITGNMSSKQMALNDVYLNDAIILTLGKLLELTGERSLVNKIKDLFS

IQRMKYKDIFARWDRRILITLMGRVTLKKINFQDESIDREIQQISKYVINNGFGNSFSCCDDMGIIEVLKHTAAILSDEKLNSSCIKTFNELVKKKIKPTINKEITYAN

ENISLMNGVVGLAYSLIRISSEKFVPKILWLE

Clostridium aurantibutyricum DSM 793 (reclassification as *C. felsineum* DSM 793 proposed)

OOL97525.1, locus tag CLAUR_41030; see also GenBank: URZ02704.1, WP_077853280.1; locus tag CLAUR_027280)

MELKSKDIVGELISKNKNRLTSISKIKSLILDNTRSIRSNLLKTERNLEIREIKYFIKEGRAIVTFTSNEKIVFQSYESKDEKIINDLIEWANKRVDKEYSLYVKKIMY

SKVYSFSEYVKPVDCKNKSELFDYYFKSGELLLILYILGCGKLKSDNIIDMEGNPILDRVRNVISSTNEVPNFNFSADEIAEKIVKDSVYNMEFLPESKKEVTEEEIYN

IKCGFEYMYNIVMCNKLELIEFLKPLLSNKLLQLSAIITNVYGLNKEDLKRQLYFLDIRFSGVKISKTKINFSFSDKKETIDRSYCISMANDFGEHMIKRGIIGVKDFV

TSRTWISTVSDEKSESYSLSPLGYSLMDGSSGIALFFAYLGCVTGKEYFKTVAIESIQNSINHINNLNNNDNVNIGAYRGIAGEIYVIWNIYKITHNMHLEDVIENGIQ
SLYILVQKSKEVDISNGICGVLGVLINIYKDKCISDKLHNMVLNLINLCSELVIKRMNYKKLELNSVFLNDSILFILAKLLETTGDERLKKYIKEILSIQKLKHSDHFG
KWDIRMLMTLMGRTILKKINFEYENIDDEIEEFTNYIIKNGFGNSISYCNEIWCIGALKYTSTILKDEKLKNSCIKTFNDFVWKEIRPLINKEINYANENIALLNGVVG
LAYSLIKMSNEEYIPSILWID

Clostridium beijerinckii NRRL B-528

OOM70943.1; CLBEIC_16210 (new NCBI locus tag CLBEIC_RS07900; identical sequences exist in strains NRRL B-593, DSM 6423, CloBei18h, DJ028, DJ030, DJ031, DJ034, DJ035, DJ051, DJ124 and DJ323)

MLSKTKPCNDVEKIINEILLNRYLTSITISIIKDFFKNIANTNIISNKEEFLRDILVHFNNYKVEESKINDYLNLATKAIRFVEKNLFQLEKEILKEKGARIKEIRVFL
DNETVIVIFTDNSKVLFFKYNILKKYSLFNEVVCYLNKKITKENNIQSRKILSSRKYCLLDASELVKEKDLFKYYFKSGELLVILYLLCCKNVNELFSLRDSIIDNNFS
ASNIAHHMLDSSVYNIDFLPLNKVNLVNFNIGAIKSGFEYMYNITLSNKSEFISFIKDIFASNLEYLGNILKKINILNEEDLKMQLYLIDAKFLTNESSIQQIILLGDE
GPCRVDRKRFIKLAGKLGDHLIKKSIIGYSNGNISRTWINFSEMKRGKKSEVSDSLYDLYNGNSGMALFFVYLGEVTQKKYFINASLEIMQESIEVINNMSGDYQHIVY
ELFTLSKIYSITKSKTIEFAINKGILFIYKIIKEGNIDNISASYVAIILSIYDVIECNKTKKIIIELGNILYKNIEFEDRDEEVLLFLIKFISITRNKEIKATIERLLT
FKGKKRLNKSHFQILLNRLKLKEVGYNDKLVDKEINEALSYIIKNEFNGSKSSYYNKDIVNIEILEYAAEVLENESLKNRCINTYNNIVNKIIEPAIYEEMCYGNKPIS
LMRGIAGYGYSLIRMCGDRRTIPILSVGGIVYMNTGKN

Clostridium cellulovorans 743B

ADL53912.1; Clocel_4251 (new NCBI locus tag CLOCEL_RS21220)

MLSNNDSAKKECIKSIPKEKHATHGISKMVARLYKSLPDIVKDKKEFITYLVEYFYENILDQDKIVEYFMFILRAIKLTEKNLFDIKSKILNTDEHIEIVKIDSYLLDG
SAIIWFSDGKSIVFKMRALEDIERFNYIINWTNSKLPDKYKLSTSCLLNYKRYGFIENIANEIPQDLEEYYFNSGQLLALLYMMDCNKVEKKDIISLRNCPSILNCTNL
FIVQEEIFNQEISSTDIAKKILDYSVYNIEFLSEDMSCLAKNYINLIKSGYKYRYNIISSNKSQLIKIINELFKGDHLILSHMIPKIYNFTENDLKQQLYFLDVRFVQF
KYQDSNFKFLVGDTYDNLNKEELKEIAIKLGDYIIQKSIIGVENSLTTRSWITDVKLGNKLELSHKCNSISYGTCGIAIFLLYLGVVTQKAYFIAASIESMRKVIYAMN
KKSIENHTLRESAEVTYTLLKIYMITKDEFIKSSVIKISSLAFSILSNSTDKLELEDMEAFTIILLAIYKEKLSDFNINRVFKIADLSYNKVLGFWKNEVCKNSLNYDV
NGVVVIFSILLYIKKNAVIEHEIQQLLKIERSIGSKKQCRKKETYKKVLISRSILKEYGYNDGLIVQELNETIKYIIEKEFGNGTYYNRDIENIELVRYAASSLEDDVL
INSCNSNLNRLVKEDLIENIKKQIKFEDRPLSFKYGTIGQGYRLIRIYNEDIVPQILFI

Clostridium diolis NJP7

OVE67234.1; CCS79_13985 (new NCBI locus tag CCS79_RS13990)

MLSKTKPCNDVEKIINEILLNRYLTSITISIIKDFFKNIANTNIISNKEEFLRDILVHFNNYKVEESKINDYLNLATKAIRFVEKNLFQLEKEILKEKGARIKEIRVFL
DNETVIVIFTDNSKVLFFKYNILKKYSLFNEVVCYLNKKITKENNIQSRKILSSRKYCLLDASELVKEKDLFKYYFKSGELLVILYLLCCKNVNELFSLRDSIIDNNFS
ASNIAHHMLDSSVYNIDFLPLNKVNLVNFNIGAIKSGFEYMYNITLSNKSEFISFIKDIFASNLEYLGNILKKINILNEEDLKMQLYLIDAKFLTNESSIQQIILLGDE
GPCRVDRKRFIKLAGKLGDHLIKKSIIGYSNGNISRTWINFSEMKRGKKSEVSDSLYDLYNGNSGMALFFVYLGEVTQKKYFINASLEIMQESIEVINNMSGDYQHIVY
ELFTLSKIYSITKSKTIEFAINKGILFIYKIIKEGNIDNISASYVAIILSIYDVIECNKTKKIIIELGNILYKNIEFEDRDEEVLLFLIKFISITRNKEIKATIERLLT
FKGKKRLNKSHFQILLNRLKLKEVGYNDKLVDKEINEALSYIIKNEFNGSKSSYYNKDIVNIEILEYAAEVLENESLKNRCINTYNNIVNKIIEPAIYEEMCYGNKPIS
LMRGIAGYGYSLIRMCGDRRTIPILSVGGIVYMNTGKN

Clostridium felsineum DSM 794

OOL89490.1; CLFE_029800 (new NCBI locus tag CLFE_RS14720)

MELKSKDIVGELISKNKNRLTSISKIKSLILDNTRSIRSNLLKTERNLEIREIKYFIKEGRAIVTFTSNEKIVFQSYESKDEKIINKLIEWANKKVDKEYSLYVKKIMY
SKGCSFSEYVKPVDCKNKNELFDYYFKSGELLLILYILGCEKLKSDNIIDMENNPILDRIRNVISSTNEVPNFNFSANEIAEKIVKDSVYNIEFLPESKKEVTEEEIYN
IKCGFEYMYNIVMCNKLELIEFLKPLLSNNILQLSTIITNVYGLNKEDLKRQLYFLDIRFSGVKISKTKINFSFSDKKETIDRSYCISMANDFGEHMIKRGIIGVKDFV
TSRTWISTISDEKNQCYSLSPLGCSFMDGSSGVALFFAYLGLVTGKEYFKTVAIESIQNSVNHINNLNNNNNVNIGAYRGIAGEIYVIWNIYKITHNMHLEDAVENGIK

SLYILVQKSKEIDISNGICGALGVLIRIYKDNYISDKLHNMVLNLINLCRELVIKRINSKKLELNSVFLNDSILFILVKLLEVTGDEKLKKYIKEILSIQKLKHSDHFV
KWDIRMLMSLMGRAILKNINFEYTNLDEEIEEFTKYIIKNGFGNSISYCNEIWCIDALKYTAAILKDEKLENSCIKTFNDFVWKEIRPLINKEITYANENIALLNGVVG
LAYSLIRMSSEEYVPNILWLN

Clostridium hungatei DSM 14427

OPX45187.1; CLHUN_10740 (new NCBI locus tag CLHUN_RS05255)

MASYDVSSGGETDLRNNKIRLNGCPDSQMADKINEFTINLPDMVQDKEAFATGLIEGYFRKPAGDVSPEDYLGFVMEVINSAAASLPEIKASVLSTKDSIKISEIRCNL
LKKSAVITFTNNSRIIYKDTMVANDIIFNNIIGWLNTRVGSEYSLYTRKLICCGSHGFLEYIQSSQCGEEELAKRFFNTGELLALLYILNCSNFKCKRIVTLAMYPVLM
NLDGIFLAHKKKSDLNISSKSIAQDIIDSSVYNIEFIPKQSKASAKLHIASIKAGFRYMYNIIVHSKREFIELLNSQFEKDSSSYLPTIITKIYGLNEGDLRRQLHFLD
VRFDAGRLNRTRVVFSGNSAVQETDREHLLDIAVRLGDYMIQKSIIGFNNFSTSRTWINTVRYGKRLIVSPKCYNLYEGNSGIALFFLYLGELTKKEYFINTAIEAMRE
PVSHIDRLDGNTLIIGAFRGISGELYALSKIYSVTRDDSIKTAIRKGLNYIGTEIDKQNRLGFFDGLSGILAVLLSICENEAFRDIRAEVLSSADLAYRKIAANIGLAD
AASGFGDGSSGIIAVLMRLLNMTGNSVVKITIEELLKMERLVITGERLQEKAGWRNGYSGILLSRLILKKSGFEDALLDEEISRAVSRTINHGFGCSPFYCNGDIGNLE
IVDYAAELLRDTGLKERCNNTFITLVKEIIEPAVNAEAKTWNKSMSLLKGISGYGYILLRRYRNNSVPQILLLE

Clostridium josui JCM17888

WP_024833747.1; K412_RS0114320

MTSYDTEGNTTQSEIMMYNYFNSEIFNVISEFVNELADIADIIEDKKAFSLSLIKNYYKINLPETQINEYLTFVMQVVNMTEKHIHDILSNILLLDEKLKIVDIHCSLV
KRSAVITFSHNRKIIFRNSSLNDEQIFNSIIRWVNFKVDERYQLYIRKIISYGSYGFLEYISSECDKQDITNSFFCIGQLLSLIYILNCKNFQEGKLLKLSRLPVLTNL
EGLFSISERKLNFAPSSKNIAQKIIDSSVYNLCIIPKRHRYLAKLNISSIKLGFQYIYKIITNSKMEFIDFLNELFDKDSPYLAAVSTNVYNLNMNDLNRQLYFLDYQF
NIKQNYKSTTKFSDNRAPNKINLNYYLELAVKLGNNIIQNSIIGVINNNTSRTWINTVECGDKIIISPECKNLYEGNSGIALFLLNLGVTTKKDYFINTAIEAMRESIS
NISTLFRCSSIELGAFNGICGEIYTLSKIYSITKDENVKKAVKLGLLYVKSAIVNQKDISLFSGLSGVLAVILAIYKNKHFSDIKVNLLAIAELAYKNILVNMDTISTT
PGYYHGINGVIIVLINLLNLIGDTAIKSVIEELLKIERNFKFDKRFSNKTMLEVEHIGILLSRVFLKQYGYDDNLIDIEIDKALAWTIKKGFGNSPFYYNGDIGALEVL
EYTAKVLKDEVLRNRCNNTFANLVENTIGPNTNDENKLWSLSMSLMKGISGYGHMLLRKCSENIPNIVLLE

Clostridium roseum DSM 6424 (reclassification as *C. felsineum* proposed)

OOM01603.1, CLROS_13610 (new accession numbers and locus tags are WP_077833301.1 and CLROS_RS21715; an identical sequence exists in strain DSM 7320)

MELKSKDIVGELISKNKNRLTSISKIKSLILDNSRSIRSNLLKTERNLEIREIKYFIKEGRAIVTFTSNEKIVFQSYESKDEKIINKLIEWANKKVDKEYSLYVKKIMY
SKGCSFSEYVKPVDCKNKNELFDYYFKSGELLLILYILGCEKLKSDNIIDMENNPILDRIRNVISSTNEVPNFNFSANEIAEKIVKDSVYNIEFLPESKKEVTEEEIYN
IKCGFEYMYNIVMCNKLELIESLKPLLSNNILQLSTIITNVYGLNKEDLKRQLYFLDIRFSGVKISKTKINFSFSDKKETIDRSYCISMANDFGEHMIKRGIIGVKDFV
TSRTWISTISDEKNQCYSLSPLGCSFMDGSSGVALFFAYLGLVTGKEYFKTVAIESIQNSVNHINNLNNNNNNVNIGAYRGIAGEIYVIWNIYKITHNMHLEDAVENGI
KSLYILVQKSKEIDISNGICGALGVLIRIYKDNYISDKLHNMVLNLINLCRELVIKRINSKKLELNSVFLNDSILFILVKLLEVTGDEKLKKYIKEILSIQKLKHSDHF
VKWDIRMLMSLMGRAILKNINFEYTNLDEEIEEFTKYIIKNGFGNSISYCNEIWCIDALKYTAAILKDEKIENSCIKTFNDFVWKEIRPLINKEITYANENIALLNGVV
GLAYSLIRMSSEEYVPNILWLN

Clostridium sp. CT7

PJI08300.1, CUB90_10685 (new NCBI locus tag CUB90_RS10660)

MELKNGNFIEEIIDKNNFRKQLAFILKIKSIVKDNYLVIKNKLLTTDEKLKVKEIKYYMKEERAIITFTNNKKIAVQTYVSEDEKIINRFIWWINEKVDEKHALYVKKA
VYSKDYSFCELVESMDCKNENELFDYYFKSGELLLVLYILKCKEIRSSNIVDVRNYPILDRIKKVFYFNNEVPNFNFSSANQIAERVIKFSVYNIEFLPKSKSNIDREC
IYQIKHGFEYMYSLVMNNKMELINLIKKLFNKNILQLSNIITSIYGMNNEDLRRQLYFIDIRFIGKTIPKTYVKFSSDKNENSINESELLSLANDFGEHMIQKGIIGVK
DFTTSRTWINTVKKDKSEKYLLRPLRNNLMNGSGGVALFFTYLGVVTKKDYFISIAIEAIQGILEHINKLCINDKINIDAFEGISGEIYAMYKLYEVTHDEHIRNVIEK
GIEILSDVIQKNNNKNTITGMCDVINVLALIYKSKGLSRINGDILNLIKICYEKVINDIKNENIKFSIIYDSIIISIVKIYEISKDDSLLNVIQGILSTQRSLKKQIYI

YDNLKWRKREFNKIISRVMLRKIGFKDKYIKREIDSTIDYIINNNFRNSPYYYGSLGSLEALKYAAYTLNDEKLKNRCVNTFNEMFRVTIEPIIRKEINFGNENISLID
GVVGLAYSLIRIEHEEIVPEILTLE

Pseudobacteroides cellulosolvens ATCC 35603 = DSM 2933

KNY28925.1; Bccel_4199 (new NCBI locus tag Bccel_RS20255)

MSSFRITEHDNIEDKIMIYGCSSKEMSKILDTFYESLPDIVQDKKNFLISLMESFYKNFTNKEQLEDYVKFILEVVNLTNSSFEEIKNYLLNTAEDIKIANIKCSLIKK
SAVITFSNSAKIVFKADPLKNNEIFNNIIKWSNSKVSADHHLNIRKMISRDTHSFIDYMQTFEYKDEKDLANYFLHSGQLLALLYILNCKDYKNISIIPQPRYPALKDV
DDIFVSMNKKLNFNISSKSIAHDIIESSVYKIGFLPGRLEPLAKTYVNSIKYGFVQHYNLVINNKMEFIDLLRFLLNSCSSCLAIITTRVYGLNEGDLKRQLYFLDVRF
PKKQIIKTPISFSEDGSFNKIEKHRLLSLSARLGDHIIQKSIIGFNGSTISRNWITTVKADKKIALSPGCYDLYEGNSGIALFLLYLGAVTKKDYFINTAMEAMGESIT
FIRNLNKNNSIEIGAFRGISGELYTLSKIYSITKDKAIKEIVKEGLLLIQTFIEKQVNTSLFNGTSGILAVLISIYENKDFSDIKNKVIATANMVYKRISSQVSCENTN
FGFGYGNDGVIAVLARFMAITGNFAIENTIKELLKKERDFNTCEMIFEKPSWQSGWPGILLSRIILKECGYNDELIDTEIYKALDYTIINGFGNSPYYYNGDMGNLEIL
EYAAEVLNNAELKNRCTNTFSELFAEVIEPYINHEKDYGNKPLSLMKGISGCGYMLLRKCSSIVPQILWLQ

LanM homologs present in species also possessing CA_C0082 homologs

Clostridium cellulovorans 743B

ADL50645.1; Clocel_0875 (new NCBI locus tag CLOCEL_RS04400)

MNNVKQLNINMKSFTQTLTIEERLNAFKENQLTTIEDTIILDEKWLNIRSLVSPQVFIEELEHMDIKKEELAFALKSFTDDEENILIEYLEKLDWFKLYKDLMKQFETI
YQNEPVFCEQLVLPFKLFVKKELYNVKNDLKNIKIEADLIEKLIDSIVSQLDKLTWKCLIVEITDYKEENILEGKDGRDRYLNFLELCYKSPTQIQLFYDKYPVLGRFV
TQKMLDLLTSVKDMILDLDVNFVGINELFNIGTNNIRDIKVSLGDTHQQGKSVAEIQFDNEKKYIYKPRNSYIEKAFNKFIEFANKNSGLKDLFINIVFYAKTFTIEQF
IEMQSCKTIVEVKDYYYRFGMLTALISLLSGSDMHFENLIAHNQYPCIIDFETFFTQVNFTHNIDDANVKVIDTQVLNLSGTGLLPMSFPMGHEGDGIDISALSGGNTK
PITRKILIAKNVHTDDMGFEYENSYILNQNNNRSTLNGEFQEYKNFKGYIYNGFNDTLDWILKNIDELKVFIKETFNDLQVRQVMKATAIYNNLVDYMDHPHYLNDMAR
IEKLLENNWAYLYSDKRLVKYEIMDMLHLEIPIFYTSTSKTYLKTSNGAYISDYFKEDVLSKVINTISNLTKEIAEKEQMKLCILLGDYSILVNERKKELAKEISKSNL
RIYQDEEVIEVCNSIAEKIMQGAVITEKSISWEYIDDTEEISKFTYLNNGLYSGRSGILLYFYYLNSIQKNEDIENFTKGLIRDVRYYKEPVENTVFKGGAGSLYTLLK
CDKDNNFKMIQDTIIFINKDNMKMDKIHWLDGVSSLIKVIDNINKTKYKSDLVLKVADGVIESICNNLDNQIVNTCSVGFGHGLIGILYALLVGQLLLKKDYNVEIEKI
LALCEEQIHNLKAPKLLDTSKDILSWSNGIVGLGIGALGCKKYTEDSRLDNYINLTIDAIKDYSFDEMSLSNGLAGELDFLASLSLIKDDPLIKEMILMKRNQMLDFYK
RNGVFLIDELIQFRNYGLFSGLSGIGYSMLRTLFPDKIPSVLTLD

Clostridium cellulovorans 743B

ADL53886.1; Clocel_4225 (new NCBI locus tag CLOCEL_RS21090)

MEKLFGNYIKKSFEIKEINKIVHRTKEYFGEFSRYYLYIFYYEFDNAIIRKYGDLFDQFNSIIQQQLDIFFKDIFYISIRVLIVEMNIMKDENKLVGANSQERYLYYSE
LLTKEEYIDYLISKYPVLNRILLEKCKNQIRLINECLSNYIQDFEPMCETFEISAQSKIKQIIVTSGDSHNGGKKVILLELSENKILYKPHDFSSEKIFNEILESINKE
QCIKYKLKTIKNITRDNYAWQDYIKAIGCTKIQEVEEYYYKIGAYLAVLYSLGCEDIHKENIIASGNNPYLIDMETLSNCQAPLINDKATMLEHFFYENSQSVFGTMLL
PTNSAVSIFDYDIGGISGDDNIETSKWEAFDIKNQGTDNLQFVKESKFITGGCDNIVKLNGEATRARDYYKNIIEGFSDCYKIFIKTPNKVVDILKESEVIIRQVLRPT
AVYSKFLEASTYPTYLTNEESFRGLFAKLDNLEEVKEKKKAQIEIDSLYEFDIPYFYSDLNTTNIYSVKGKVDNYINYSVMDAISGKARKFSEKDLKVQIYYINLALST
QPNTSEMIYKYNLFSKSQNLKNITINHIAKQIGDVIEEKLIWDYRKQSCFFMIDTVVKEKRKYCSMDSSLYGSGGVILYYLSLYKATCDLKYKHIVEGLINGHIGLHSK
KSLEEGIGLFSGLTSLAYIYYQCYRVLGKKQYLEDVLEVLKEIEENLHMWDELDLSTGLSGVIIFLSELYKTENNEQYLLIANGLADKLYKLVLKANFNMLTGLAHGYA
GVAWALYSIGHIVNNEDYINCAMKCIEEENMYYDSEKNNWKDKRNGSSESFYWCYGAFGIGIARLKMYEIAHNDILLKDIEKCRYIYINYNLINSKYNHSLCHGLTGNL
SAIKMFSDFYKNDNKLKKKYKEMLDILLNDVVNGKVIWGDKELIEDYSFMIGLSGIGYELLRHEATNSVNILALEV

Clostridium cellulovorans 743B

ADL53889; Clocel_4228 (new NCBI locus tag CLOCEL_RS21105)

MEDMISYYEKCWMKLYPEMKSIEELNTYLKKVFGKSLKEKLQNERVEKVSVKEYNRNLKMFLDDNMLENMRFSRFYGPIMVEYIENLPKYIEKTMIVKNIKLFMESMIL
QLSDLMCSIAFRTMVFEINNAKNKNLLKGESPEERYKYFNNELLDDYQYRKSLYSEYCFLVETLDECAKNFVKYIEEILVNTSKNMCRIQSDVNSNIELGKLINIEFAL
GDTHCRGKSVAKLIFENTIIYYKPRNSIIDNKFQSVLNLINEKGILSGRKYRVMNIHGTSECGWFENIKYEECRSIDNVHDYYLKIGGLIGILYFFNATDFHHENIIAC
AENPMLIDLESIFSVEMKSKVFDENSAYNNAIEYLKSSVQSIGILPNKLHIGDLDDKYETGGIVYKEKQVAPIKSLKVVNDASDGIRTELVNSIIEGNLNAPKYNGNII
NPKEYVEDIKEGFRLVYKWILGNKKEFIEFVETSFSETKIRIILKPTFMYAQINSIAKHPNFMSSEDENELINARIGIYADNIDIIKSEIRSLKRYEIPYFSALFNEEK
LFDEDENVLESRLIISPQLLFRNKVCKATEIDLNNQIDFISISFLSKNPEELRTGIHYVEDAVEIINTDSYLNVAKEIGDYLYSIAIIGENQHGKSDATWIGSAVSKID
VNDWTYSVSDLDLYNGNSGIALFLLNLWKVTKDKKYLDLAIQAAELIISIIKNKTFNHSTLIGGFNGIGSYIYIISKLVVNTNDEYFYSTLIESIDLLEERIEAASEMD
LVAGASGMLAVLLNVYSEIDDKLIKEKVKPLLYMLFYKIQENVKSGGKLIRYSGFGHGIAGCIPYLYKLYLIDENREVYQLFSELLSYERDHFYSKEEKDWVMSDDEVN
YSKAWCHGAPGILLEKIILKELGYEDEYLDQEIKVALNNIKKKCIGNNIVYCHGDIGNLDIIQYAAKISKDEKMIKECSNTYDKLFQLHIKNNWNSEASAYSKCKGIMV
GVSGIGLSLLRMINKYDIDDFLWLS

Clostridium hungatei DSM 14427
OPX45176.1; CLHUN_10630 (new NCBI locus tag CLHUN_RS05205)

MSNSTVYKQGLITTTRRDEIISCWKSLFPMKSRQYNFEDFLEKISGKKLDFFLENYDSNEVSEEIEQVFSHMEENRHLELLEEILSSQAKKNMWAFFFKPVLLLYMDNI
TELAGLDIIEDKDSFLDSVIDSIIEKLTVLSYRTIIVETNIARLDQRLEGETSEERGKYYTQHLLRDMNYLKEIYLVYPELYEAMKRTALYITMYYREIILNTKENLKE
LEKIFNGGNSLGRLKNIKLGNGDTHNNGKTVATLSFVSKMLIYKPRSFTMEKAYEEFLSWVNGRIPGFSPLSACKTYGMGDAGWMEFIQHSECKTMEEVEEFYLKMGEL
LCLFYTLNSKDCHCENIIACGKQPILIDLETVLHTDEYDKHQEFASIEEYIGNYIQNSVDSVMILPSLLQNFNNGEVMEIGAIGSGKPKKSPFKTQKIKNFDSDDITVE
EEYKDIADSANYPLYKGEKIGGNGYIGFVRQGFVNTYEWILENKAEYTAKIKDLFGKVECRVIYKATNDYNQLIATSYHPDLLHNRMDRLVYFHRIGILIPDIENFHEQ
KLYRTEIEAMLNGDVPSFKITADSLRATNHKNEVVYDKYKKSIIETLEDKINRLSKIDLERQVALIYMSYMGCKMETDLPRGTKTLFSKSGNSGDNYDYLKLADSIGEK
IIKRGFSTWVNGKREHSWICYAGFGDDYYSINPVGWDLYKGNCGIAVYFMYLGKTLKEESYIQYAKDTLNSVDRMINLNNCRDIETMGYGVFTGIFSYAYALYRFLRAG
VFGKEEAEAAWERIYRIIRYTNEKIQEQEKVEVLGGVCGIAGVLASIYPSVSQTQQGTVKELISKIIARLKKAAVYISPEEITWTQNGDIGYAHGNAGIMCHLARCYEI
IPDREILSLIEKCLNYERNIRFDKSAKRWLVREKTHYFSWCNGIAGLLLSKLLLYQSPARDEKLLEEINRLMEQLKLYGFGTDYSICHGDMGSICILRYAAELTGQSRL
LKQCTYALDNYIRNQMLAQADSMYTIEDWGLMVGKAGIGMGLLDVFNQNNLTAELLSLR

Clostridium josui JCM17888
WP_024832967.1; K412_RS0109925

MSERDVSAHFENVIAHGEDPVLVDLETLFYPKVKIQSARNKDSGFQAANSFINKSVSKIGLLPTRVRIKRDNKIQSVDVGALSESKNQSSILKSLVLKNTQSDNLCLSY
EYLSIEQKKNSPKLLGEALDPKLFMDDLIYGFQAFYKFVQNAKSEMIAYTMSLFSECQIRVILKSTVTYTSLLNIASHPDFMRHPIHRVILLSKIGANDYYNIYIKRFE
LSELCRNQVPYFYTRFKSHDLKGYPNVCFPNVIERNTPEMLVDKVLNLSTKDMKTQISLIKDAFFVRDPEDDITGIKFNYKQNIEVNPNEWLTLSQQIAEYLCSRSFCG
INDDGWADRAYIGAQTLMTDTEEWNKDVDGLDLYDGNSGVALFFTYLWKVSGLEKYLNYAIDTIQPVVQIVNHSDEIDYKGAIGAYKGLGGLAYSLNKIALLTKDLSLS
HAVESVLDLIDKNIYHDINYDLVGGGIGCLAALLSIYQKPYNQTVKNRSLSLAIKVYEFLKNKFVKVEYGKVINLDKERISSGFAHGTAGFSPYLYKLYTITSNNEIFE
LFQSILAYERHIFYAPEMPGGHSSLKKGEMDNNWCNGVSGILLSKLLLREFGYHDELLDMEIKLAYDLTIKNSFGYNLTYCHGDLSALTIIRYYAKTMNNLQLESKCIN
VFQQLFDRIKDNWESEKKTINKFNGLMLGASGLGFAMLKQYDFDNVDEFLWLV

Clostridium josui JCM17888
WP_081741753.1; K412_RS0109930

MVGLITSVRMEKVMKKVENVLESRYFENLGSSNLVDKICSNLSAAFGNIHRPFLLYCEKHIEVSLDKFSLNYSIKVDKEVIMSSVLASVAEEITALTIRILIIELNCLK
SNNELQGKDSTERYNDFNQKLSDVSYLIAFFKRYPVLLYLIDTKITGRLQLVDELLERLGEDKQSIESTFNICMHSLTNVNISSGDSHNNGRKVTILQFGERYLVYKPH
GLSPESLFNQIVDYINKKASFHCKLKKLKCIDCVNYGWQEFAVYQKAENNEDIYRFYYRTGALLAIFYMFSCSDLHHENILACKDSPAIFDLETLVNIFQQSIEGGNTN
ADISREFACSVLGTMLLPANFVNGAFDFDLSGLAGSDDSVSRKWFYFQVKNAGTDEICLNKEPCTSPRMKNALMYKDEIVSPKLNVLSIQNGFLDCYNVIENNRNEILD
IICSNRIVIRHVLRPTAVYARFLEASTQPNYLENMEARKALFSKLYNNNTQLDIVKCEVEALLKHDVPYFSSEIDSCTLLGNLVEKIPEYFQKTAYQVIEDKVKQFSNN
DVNKQLYYISQSLSTLKEHVTNYSYNFGMSGQDSYLSNAKKIADQIYNLSVVNSEKDEASFLMTLELPNMRRLISSMDFNIYTGGGIIILFLAVLGQELKEQKYTRLAEQ

FLNYQLSSAEQQKSLSAYTGIGSGAYICYNMFKLTNRSIYYEKCRRLVTEINFVDNCPNDFVGGISGLIILLLNIFEKEQDSLFIEKAEWLGKELYRSLRSENQDLITG

LAHGLSGYAWALIKLGKFTNNQTYIDFGLELIRKENSYYEPSEYNWRDLRDDHQFLSYWCYGAAGIALCRMKVLELINQADDVLTNDLQNGIESIKLNKAKSHSLCHGS

FGNIDILLEIGKSRHMDRWVSLAEDIAKDELADIQKNGVRFGDVASLSDFSFMQGLGGIGYALVRLANHNYPSILSLDVM

Clostridium roseum DSM 6424 (reclassification as *C. felsineum* proposed)

OOM02070.1; CLROS_09680 (new accession number and locus tag are WP_077832904.1 and CLROS_RS19500, an identical sequence exists in strain DSM 7320)

MNTLKQLNIINTKSFIYALTINEKLNAFKENKLLPTEDTILLDNKWLNVRSLVPPKTFSEELKHLDITREELSFTLKSFTLYEENILISYLEKLKWFKFYKTLMAKFET

NYQSKPVVCDQLILPFELFVKQELNNIQINLKNIKIDVGLLKKLRCNITNQLSKLTFKCLIVELTDYKELNLLEGKDGRDRYLNFLDMCYKSPDQIKSFYNKYPVLCRI

IIQKMLDLLTSLKDMFLNLDSNFVKINSLFNINTNIIKNIEISLGDTHQHGASVAEITFHNNKKYIYKPRNLYIEKAFNTLLEFINKNSNLKNLFITKAFYGKTFTVEA

FIEVQTCNTLEEVNNYYYRFGMLIALISLLNGSDMHFENIISHNQYPCIIDFETLFTQFDFLYNSSNANNKVLFNQVLNLSGTGLLPIGFRLPNMTDEIDLSALSVSTT

NPASREMLVAKNIYTDDMCFVYENVSISNISKNKVTLNQKPLDYKTFKQYIYMGFNATLEWIFENIKILKPFIAKTFSNLQLRQVMKATALYGELIDYMDHPHYLRDMI

KLEKLLENNWAYPYADLRLIKYEIMDMLHMEIPIFYTNTSETYLKTSTNEYISKYFNESPLAKVLNKFSKLTEKAVKKQELKLQLLLGDYNFLVNQRKKLLIKSISKNT

LRKYQDEEVFNTCISISEVIMKSAITNKSSVSWEHIDNSGNFPKLTHLNNGLYSGRSGILLYFYYLNSVYKNNSISTFTDYLLQDVKRYREYDKDILLDGSSGSLYALL

KCEKDNSKFDVIFDNIIFSCNNINFENNISWINGSASLVKLINFVNETKYKTPIALDIVNKILESLSTSLNNKIIKTYNLGFGNGLMGLLYSVLIGQALLKKDYGKEIE

KLLNLCEEQLQNFKTNESNQPLSWNNGIVGLGIGSLACKKYIDIPNLDNYINLTTDILRNSQFNDMSLYNGLAGELDFLVSLSLNKNDSTINQLILKKRDYIVDFYKSN

NSILIDELPEFRNCGLFSGLSGVGYSLLRTLFPNQLPSVLILD

Clostridium sp. CT7

PJI09179.1; CUB90_15435 (new NCBI locus tag CUB90_RS15410)

MELNLAKTLTNEQKIDIYKGNLLKEKQVNEVINDWIKNDNLLSYNNLLKKLSIEGINLQEFASVLNKNNISIDYKKISSTKWYELYKNIIQQYSKSDNFNFYNEESIMY

FLNIMIYWCKVQMMQQISKYNNIKIHQDIVNIVLNSIGTNLFNVASKTVIYEYYNQEEKNFRDYNLSNFKNIKGIENFFSKYPVIAKRLVTKVKYLLENYLSSFNRIDR

DFEIIESTLGIEIGNIITNIEGNMGDTHEKGKFVIQYEFNNKTKFVYKPKKLAMAKAFYKIVAWINKKSDCEKFKIPKNYYNEEYTIEEYIESKTCETEQEIKRYYKRL

GQFLGIVYQLNGNDFHYENVIANGEFPYLIDLETIFTQPIPLNKRDIFDYYSIIKNQDSIINTCFLPSNTGLADEKGKGIDISALSNKKQKLPYKILSLVGDIDNPKFE

YIDFEVGTKNNIPLLNGEKVNYENYKSCIEEGFTEISKFIIENKFEFLKQLNMFQGISVRQLIRNTNDYFKILEYASHPKYTMDMASLEKMLYFMWEYNLDDKRCITSE

IEDLIRDDIPLFRTITTSKDLIDSKGKIIKNYFEKSAFNRVKEKIINFNKNELNKQLDYLNISLNNSSNVINRKSRMKNNLYEFNKTLLKDDGYINSNIMLNDINSIIE

SFFIKYKDECICPNLINGDVKLDFGFKKGLIGIYYYLYMNNSLDVNIQKIRNVFSNEKIISLSISNNKLQDLLDLLNIIERTFDNNYNEDVLTLRSNIIKILNEELINN

KVNLLDTIKFIKIIGELYKTNKSYELKKTILKLKNNLEIKLLQEGLITLKYNSHISTIDLLECIDLIKDIYPNEQFSFNLDYINKINSEYFEQIYNLESLKMYSNEEYR

NIIRSLSINLNNTYIERYNLKQCVFKVVNENEIDENIICILDILISCYTKSEDKEIKSLLDKKFKQLVQYYTITNNYPVDEMNYFKNLSIGNGISGIGYEILRYRNNKI

PDVLNLF

Pseudobacteroides cellulosolvens ATCC 35603 = DSM 2933

WP_036937461.1; Q332_RS05130

MNNSLQYNQECKKIIEEDKIINYWRSLFPEKKRQYKIEDLLGKISGKTLDYFLQNYNKEEEILDEIDKTFSDLNESCYTKILDEILLKQTKKNMWSFFFKPVLLLYMDR

LMKLAQSSDILEDGDLFLENMIDNILDKLTALSYRTIIVETNIAKLDKRLKGETSLERGKYYTHNLLRDMDYLKEVYFVYPELYKVLKRTILYNVCYFEEIISNIKSNL

NELEKTFKDGKSLGKIQSIMLGNGDTHNNGKTVATIKFSRKVLMYKPRSFSMEKAYDSFIKWVNNRIPGFSPLNTCKTYGIKAAGWMEFIEHSECKAMEEVNEFYLKMG

ELLCLFYTLNSKDCHCENIIANGKYPILIDLETVLHTDECHKNEEIDSVEAYIDNYIQNSVNSVLILPALLQNFNTNEVMEIGAIGSGKAKKSPFKTQKITNFDSDNIS

VENVYKEISDAGNYPLYDGKKVGGNGYIGYVRQGFVSTYKWILENKDEYIDKIKELFNNVECRVIYKATNDYNQLISTSYHPDLLHNGMDRIVYFHRIGILIKDIDNFD

EQKLYQTEIEAMLNGDVPSFIINADSHKAINHKNEVVYDKYKKSIIETLEEKIMKLNAIDLERQVALIYLSYIGCKMETDLPDKTETQFSDGDDCGVNYDYLKEARNIG

EKIIERGFSVVVNNKKENSWICYTGFGDDYYSVNPVGWDLYKGNCGMAVFFMYLGKTLKKESYIQYAKDTLNSVDRMININNVKDIEAMGFGVFTGIYSYVYTLYKFIQ

YGLYNEAEIKEVWERIYKIFEFTNENIGKQDRIDVLSGISGIMGVLTTIYKTLDIKYQEVVKEVLSKIVDKLKKEVIYISSEEISWTDNGDIGYAHGNAGIMCHLAGCY

EILSDPDILEMIYKSLNYERNTRFNKNDNMWMIREKAHYYSWCNGIAGLLLSKIMLYQSNAKDEKLIGEIKLLIEQLKKYGFGNDFSICHGDIGSISILRYAAEFMNDT
QLMKQCTYTLNKFIQNYLVEQAQSLYTLEDWGIMAGKAGIGLGLLDEFNHNNFIAELLSFK

LanM homologs identified in blastp searches as being most similar to CA_C0082 homologs and other LanM homologs present in the same species

Bacillus amyloliquefaciens Y14

APB83634.1; BAMY_16020 (new NCBI locus tag BAMY_RS16385; an identical sequence is present in *Bacillus velezensis* 7751-1)

MDISYENNNFITDAVINFSSPTNEVEKLRIKNKDYFGNFYTFFLDFYQQRLVNTLENASKENNVKLNKHKIISSALEAFNQELIQLCIRTLIVDINERKEKGLLEGKDS
RSRYKNYNNLIFKSEYVAEILNKYPVLTYLISSRISNKILYLKEVLENLRKNRQDIYRELKIEFDEVSNIYFSSGDTHNGGKNVLIIETNQGKIVYKPHSLSPDILFNS
IVDYVNNSDKILKKIYKTRTLNYKDYGYQEFIDYKECETSEKLNFYFYRVGVSLSIFHIIGCDDLHHENLIAHGEYPVVIDLETLIKNNSMYKPRNNNLIDNFHEDINY
SVLGTMLLPLNLQTSIFDFDLGGISNDENQTSEIWKSYIIDFEGTDEIQLTKKSVIMNSTQNRAAYNGKAADPKDYIEEILKGFTDCYNFVLENKSGFYDLVKKVGNSN
LEVRQVLRATSIYARFLEASTHPNYLSSFEERKELFKKINIAEGVTDKFSKKNLNELESLMCNDIPYFSTMYNSLDLICNKSTSIVNFFRECLLDVVLNKIKSISKTSL
KKQQYYIRMSLTTTIKDSWKRTNKHNKKYRPKLFGNNYKNYLECATEIGDLFLETAIWNNDRSKCTWVTPIISENNKVKLGPLNFDLYEGGGVILFLALLGKETGNKEY
FDLAFAGMRGIEELFLSDDKMDGRLSLFTGIGSLSYIYYHLYTYTNDYKYYEKFKKYINKINEMNISGDIPLDIVGGVSSLIVFLLNLYKETKLDILNSVCCKLGNTLY
QCLEKNGKHNYLTGLSHGYSGFTWALCYLGHITKDEKYTTLGKELLKRENKFFDLNTSNWKDLREGEGNSDPVYWCHGAGGIALSRAFLKNLLKNKENVVDKEFDKDLN
CAISKLLSDGFKKTTDHSLCHGIFGNIDILLKLSELLNDIDLQEVAFKEAQNAINYIRNKGFIPGLQDHFDLNTFMLGLGGVGYSLLRLHNPVNPSLLAMEVRSYNE

Bacillus amyloliquefaciens Y14

APB83636.1; BAMY_16030 (new NCBI locus tag BAMY_RS16395; an identical sequence is present in *Bacillus velezensis* 7751-1)

MKTNKYNLQYWMKMFPEVNNKDELQKIFLNVSGKTIQSLIRDDTNIEQNTNIIDIDRVLDHKKSDFLVRNSEEFLEHNPFFHFFSPFLNCKIEEFIVKVRIEDAVDNED
EFVKKVIKHIVDLMFEKAFRVLVLEVNIARLEGNLEGTTPQERLNHFLVVSLNDESFLKSVYKEYEVLTSLLCVTIDDYFTYVMEIIKNTKREISSLNSKFNSDNDLGA
ITNITTGLGDTHQKGKSVSTIYFKSGKKIIYKPRDLRLEQGFQEVLYWLGGKNIPGILNFKRVQIHTVNDSGWMEHIDYKSCFNKKEANDFYTRSGNLLCLLYLLNSVD
FHHENLIAHGSFPVLVDLESLFHARLKVDQIDKKSAVVKATELVDNSVQSISLLPTKISKRVGDKDISLDIGGLGAHKEQLSPHKSLVIENAGTDTIKILRKNTFIKPQ
LNNPSIKTGSYLYSENYTGQIKDGFASLYSWVMLNKDEFWNKISQTFIETNSRFIFRPTYLYTQLLRISSHPDFMRDTYRRKIILHRIGIDYIQEYKDILNSEYKDLLT
GDVPFFTSSIEHENLIDSRGEKIHSILEEPPIKTVKQKIFNLSKEDLKRQIDFIEMSYISNEKRLKEVTDIKFSKAANLNKIKSENWIDEATQIGEFIVDNSVCGINKK
QKDRMWIGPSLEGIEEDIWNANVLGFDIYNGNSGIALFLGYLGEILNRKDFKQAAIETMRPIQKFISEIKEDHPYLIGAFQGISGYFYTLNKLSNLSEDSELKGASLEN
ISVLSKLGKFDKVYDLIGGSLGSLAVALSIIPDITEENSKKEILKISHTHCDHILSVAKNFEEQISWPGKFSAAYSGFSHGNSGFIAYLYKYFKLTNDEKLLEVIQKAL
RFERRLYSEDHNNWYTTENKDKLANGWCHGAPGILLSKLILKDNGFEDEYIEKEISTAIDSSIRNGIGNNPTYCHGDLGVLSILNYASDLTNNINLKNRCLRTYQDLFE
NVLTKKWRKRDLVCTRSYSLMIGLSGIGYSMIKNYAPEIVPNFLWLE

Bacillus cereus VD166

EJR73612.1; IK9_05147 (new NCBI locus tag RS26410)

MTNNLETNELEITFDDVINYWMTLFPEITDKKDLEKLLLISDTSFKELEKKYNEESLTNIDINRFFEMKIDKRVEKVFDKLIDDLPWAHFFKPIVNYYAKDLYRFLTTT
PIIEDTDNLFEQIIGGMVQRLDQISYKVLILETNIARLENKLVGNSSQEKALYFRNVLLNDASFVQALYREYQELSNIMDFKVKHTFDYMQEIIKDTTNQWDHLSKIFT
KENNLGKLREITLGAGDTHNGGKSVSILKFSCGKNVVYKPRDLQLEKGFYNFIQWINQQERENSLDLRAAKVHFIEHAGWVECIEYKTCETSEQIKRFYYRTGQLLGIL
YAVNGKDFHYENIIAQGEYPILIDLETFLHPDLHTVDKNNASASMIANEIISSSVDGIGLLPSPIINNKTNKSVDVGGVGAEEVQVSPFKSTFIADIDSDQVRITKDYG
LLGTQKNNPNLNGIVLKSKDYLEQTKNGFIDTYRWILKNKDLFISKVVELFDQKECRVLFRSTYIYAQLLTTSYHPDLLRNPLDRKVYLHRLALILTDEFKDIGLSEIY
DMQQGDVPLFTSTTSQNKIYNGRKQHIVGPKIVRSPIETIKWKVEKLSEEDLERQLNFIDITYLTKSNDSSKDITNLKLQEHSDSEITQNEREKWLNLAIEIGDHLLDK
SIIGKYDGKIDRTWIGSFSADKESTTTYLTQVGQDLYGGNSGIALFLAHLGFITNSQRYKEATYEAILPVIQSVELFEGTTNVGIGAFTGISGQIYAIFQIGHLFKDNQ

LIKLAQEKILLLEKIINPKTIHDVIGGIAGTMAVALSMYENSTDNIIKKDLINLANHCFNTLKESTVQFNERDGITWGEEGYTGFSHGNAGITAYLAKLYNITKNAEIL

PIIEESLKYEKTLYCEETNNWYNSIEKENRAYGWCHGAPSILLNRVMLYQYKCCNELANRDLQIALETTKNDGLGRNPSLCHGDLGNLRILHFASTVLEDKILQNQCIA

TFDELYTSFLQHKWDKGVFRGTENYGLMVGLSGFGYTCLQFYAPEVVKDILWLN

Bacillus pseudomycoides AFS080276

PFZ09488.1; COL63_22295 (new NCBI locus tag COL63_RS22300)

MTHTLETNELEITFDDVINYWMTLFPEITDKKDLEKILTSISDTSFKELEKEYNEASLTNVDINRFFRAPLDERVEKAFDKLIDDLPWAHFFRPVVNYYAKDLYSFLTE

VPIIEDTDNLFEQIIGGMVQRLDQISYKVMILETNIARLENKLVGNSSQEKALYFRNVMLKDASYVQDLYREYQELSNIMDFKVKHTFDYMQEIIKDTNNQWDHLSKIF

TKENNLGKLREVALGAGDTHNGGKSVSILKFSCGKNVVYKPRDLQLEKGFYDFIQWINQQDMENSLDLKAAKVHFIEHAGWVECIEYQTCETSEQIKRFYYRTGQLLGI

LYAVNGKDFHYENIIAQGEYPLLIDLETFLHPDLHTVDKNNASASMIANEIISSSVDGIGLLPSPIINNKTNKSVDVGGVGAEEVQVSPFKSTFIADVDTDQVRITKDY

GLLGTQKNNPNLNGIVLKSKDYLKQTKRGFIDTYRWILKNKNSFISKVVELFDQKECRVLFRSTYIYAQLLTTSYHPDLLRNPLDRKVYLHRLALILTDEFKDIGLSEI

YDMKQGDVPLFTSTTNQNKIYNGRKQHIVGPKIVRSPIETIKWKVENLSEADLERQLNFIDITYLTKSNDSNKDITNIKLQEHSDTEISQNEREKWLNLAIEIGEHILN

KSITGKHDGKTDRTWIGSFSADKESTTTYLTQVGQDVYGGNSGIALFLAHLGFITNDQHYKKASYEAILPVIQAVELFDGKTNVGIGAFTGISGQIYTIFQIGHLFKDD

QLIKLANEKILLLEKIINKKTLHDVIGGIAGTLAVALSMYEKSTDNIIKENLINLANYCFNTLKESAIRFNDRDGITWGEEGYTGFSHGNAGVTAYLAKLYNITKNTEI

LPIIEESLKFEKTLYCEKANNWYNSIEKENRAYGWCHGAPSILLNRAMLHQYNCCHELANKDLQIALETTKKDGLGRNPSLCHGDLGNLRILHFVASVLKDETLKNQCI

ATFDELYNSFLQHKWDKGVFRGTENYGLMVGLSGFGYTCLQFYAPEVVTDILWLN

Bacillus thuringiensis AFS059517

PFW00888.1; COL21_07440 (new NCBI locus tag COL21_RS07435)

MTNNLETNELEITFDDVIDYWMTLFPEITEKKDLEKILSISDTSFKELEKEYNEASSTNVDINRFFRVQIDKRVEKVFDKLIDDLPWAHFFKPIVNYYAEDLYGFLTKI

PIIEDTDNLFEQIIGGMVQRLDRISYKVMILETNVARLENKLVGNSSQEKALYFRNVLLKDASFVQPLYREYQELSNIMDFKVKHTFDYMKEIIKDTTDQWDHLSKIFT

KENNLGKLREITLGAGDTHNGGRSVSILKFSCGKNVVYKPRDLQLEKGFYDFIQWINQQERENSLDLRAAKVHFIEHAGWVECIEYQACETSEQIKRFYYRTGQLLGIL

YAVNGKDFHFENIIAQGEYPVLIDLETFLHPDLHTVDKNNASASMLANEIISSSVDGIGLLPSPIINNKTNKSVDVGGVGAEEVQVSPFKSTFIADVDSDQVRITKDYG

LLGTQKNNPNLNGIVLKSKDYLEQTKNGFIDTYRWILKNKNSFISKVVELFDQKECRVLFRSTYIYAQLLTTSYHPDLLRNPLDRKVYLHRLALILTDEFKDIGLSEIY

DMKQGDIPLFTSTTNENKIYNGRKQHIVGPKIVHSPIETIKWKVENLSETDLERQLNFIDITYLTKSNDSNKDITNLKLKEHSDTEISQNEREKWLNLALEIGEHLLDK

SIIGKNDGKIDRTWIGSFSADKESTTTYLSQVGQDMYGGNSGIALFLAHLGFITNSQHYKKATYEAILPVVQSVELFEGTTNVGIGAFTGISGQIYTIFQIGHLFKDDQ

LIKLAQEKILLLEKIINPKSIHDVIGGIAGTMAVALSMYEHSTDNIIKKDLINLANHCFNALKESTVQFNDRDGITWGVEGYTGFSHGNAGITAYLAKLYNITKNIEIL

PIIEEALKFEKTLYCEETNNWYNSVEKENRAYGWCHGAPSMLLNRVMLYQYECCVELINKDLQIALETTKNDGLGRNPSLCHGDLGNLRILHFAASVLKDETLQKQCIA

TFDELYTSFLQHKWNKGVFRGTENYGLMVGLSGFGYTCLQFYAPEVVKDILWLN

Clostridium beijerinckii NCIMB 8052

ABR36694.1; Cbei_4586 (identical sequences are present in strains SA-1, 4J9, MF28, NRRL B-591)

MELNLAKTLTTEEKISLFKGNLLEKYKANKVIDEWIKNDNMLSYDNLIKKLELEGIDFEKFATIVSEKEIDNQEVSYAKWYELYKNIISEYSNVQNYNFYNEESIMYFL

NAFIYWCNVQLNKEICKYNNIKIHSNQIDGILSNIGKNLFNICSKTIVYEYYNQEKQNFKFYNVSNFKNVSDLQKFFAKYPVMLRRLVVKVECLLNTYIESFFRIDRDF

KEITEKLDIKLGNIITNITGNLGDTHEKGKFVIKYEFNNTTKIIYKPKDLNIAKKFYNIISWINKNCDCEKIAIPNNYYNSDYTVEEYIEATPCENEDQIMRYYERLGQ

LIAITFLLSGNDFHKENIVANGEFPYLIDLETLFNQPITIQAKDIFDYNNLRIQDSINRTSFLPSNSGLADENGNGIDISGLSYKDQKLPYKILKLMGGVDNPRFDYVE

CEIKAQNNIPILDKQKIGYIKYKSCIKEGFLKLSKFIIKNKFEFLKQIEVFKNTKVRQLMRSTINYARILEYASHPKYTVSMLNFQKMVYYMWEYPFNDKRLITSEVED

LLYDDIPLFKTITTSRDLIDSKGRIIKNYFNKSALDHVKERIINFDEKSVNRQLDYLNIAINNSKNVIDKKLRMKNELYKFNKSLVKDVKCENLDILLNDINQLIEYFS

IKYENQCIWENLINGKIKLESGYSKGILGIYYYLYMYNNVNGNSSNNLKEIIAVFSNEKFLNLGLFNNKIQDLLDFLSIIDCTLGNTFNTDILNLKSRINEVLNKQLDN

GKLSLSMTIKSIKVLGKLYIVNKSYDLKKTISKLKDNLEIQLLTEGLTNLKYDNDVSVIDLLDCIILINSIYPNKEFNFNLEYIKELNNKYFEQIYKLESIEFYNNKEY

RNIIRSLSLNISNNHIEYNQLKRFVLKIIKEKEIDENIIFIIDILINLYLKNKDQEIKKVLDEKMKDLEQYYAITNNYPIDEMNHFKNLSIENGLSGIGYEILRYFNNR

IPSIINMI

Clostridium beijerinckii ATCC 39058

OOM50278.1; CBEIJ_06930 (new NCBI locus tag CBEIJ_RS03400)

MELNLAKTLTTEEKISLFKGNLLEKYKANKVIDEWIKNDNMLSYDNLIKKLELEGIDFEKFATIVSEKEIDNQEVSYAKWYELYKNIISEYSNVQNYNFYNEESIMYFL
NAFIYWCNVQLNKEICKYNNIKIHSNQIDGILSNIGKNLFNICSKTIVYEYYNQEKQNFKFYNVSNFKNVSDLQKFFAKYPVMLRRLVVKVECLLNTYIESFFRIDRDF
KEITEKLDIKLGNIITNITGNLGDTHEKGKFVIKYEFNNTTKIIYKPKDLNIAKKFYNIISWINKNCDCEKIAIPNNYYNSDYTVEEYIEATPCENEDQIMRYYERLGQ
LIAITFLLSGNDFHKENIVANGEFPYLIDLETLFNQPITIQAKDIFDYNNLRIQDSINRTSFLPSNSGLADENGNGIDISGLSYKDQKLPYKILKLMGGVDNPRFDYVE
CEIKAQNNIPILDKQKIGYIKYKSCIKEGFLKLSKFIIKNKFEFLKQIEVFKNTKVRQLMRSTINYARILEYASHPKYTVSMLNFQKMVYYMWEYPFNDKRLITSEVED
LLYDDIPLFKTITTSRDLIDSKGRIIKNYFNKSALDHVKERIINFDEKSVNRQLDYLNIAINNSKNVIDKKLRMKNELYKFNKSLVKDVKCENLDILLNDINQLIEYFS
IKYENQCIWENLINGKIKLESGYSKGILGIYYYLYMYNNVNGNSSNNLKEIIAVFSNEKFLNLGLFNNKIQDLLDFLSIIDCTLGNIFNTDILNLKSRINEVLNKQLDN
GKLSLSMTIKSIKVLGKLYIVNKSYDLKKTISKLKDNLEIQLLTEGLTNLKYDNDVSVIDLLDCIILINSIYPNKEFNFNLEYIKELNNKYFEQIYKLESIEFYNNKEY
RNIIRSLSLNISNNHIEYNQLKRFVLKIIKEKEIDENIIFIIDILINLYLKNKDQEIKKVLDEKMKDLEQYYAITNNYPIDEMNHFKNLSIENGLSGIGYEILRYFNNR
IPSIINMI

Clostridium frigidicarnis DSM 12271

SFB45753.1; SAMN04488528_10694 (new NCBI locus tag BM119_RS18780)

MYKDYYNDVEKLYEIIRNSNEKSDEVFQICKKNNELFGKFFIEFLDFFKVKLDDILYKFRKNNCIELIEKNIIISVLNSLIEDLLNVSGRTLIAEINIKREENLLIGET
SKSRYNFFNELLLNDKYKIELLKKYPVLAYLIYTKIFNKLEVISELLERLVRDYAIIKSELNMDFKVIQNINFSSGDTHNGGKSVLIIVTDKGKIVYKPHSLSPEKMFG
EIIDFINKKETLKIKVNKIRTIDFNGYGWQEFAEYKSCKNLTEVCNYFYKIGVELAIFHMLGCDDLHYENLIACGENPVFIDLETLLKNNSSDTLYSENLTTVFNQNIN
DSVLGTMLLPLNVKGSVFDYDMGGISSYGDQKSNFWKSYVIENAGTDEIRLNKKSINIGDSYNKVMLNEEVMEPIDFNQDIHNGFFDCYRLILDNKTDLIKFINLIKNV
EVRQVLRATAVYARFLEAATHPSYLTDFKEREMLLSKIRVLNQETTENLKNKNLYEIDALMDNDIPYFTTYLWTNDLVCNNKVKIRNYYNSTLSEILLDRVQSLSEINL
KKQLYYIRMSLATTVKDIWKKESLKENSNITYFKNKKSYIECAKELGDLFCETAIWSKDKNTCTWLTQIIAKEDKFELGPLNYSLYEGGGVILFLNTLAKETGESKYSK
VSMAGMKGIEEIYLQHSTNQLQPSLFQGIGSLIYIYYSIYSLNDDKDAYSKYIKYLKELSEYDIDKTALDVISGVSGLIIVLLNIYSKEKSELLLTTCDKLGKVLYESL
LEDNLNHFTGLSHGYAGFSWALILLGKYVNNNNYLELGKKLIEIENEKFNKDKLNWKDIRTEDKNVDHVLWCHGASGIALSRANLLNCLDDKDTIKIQRDLELAIKKLT
SEGFTDELDHSICHGIFGNIDILLKLSQILNDRMLLEQAYKEADKALKYIRTKGIKCGMKKSFDISTFMIGLSGIGYVLLRLHNPRYASVLSLEVLNAEVL

Clostridium saccharobutylicum NCP 165

OOM18158; CLSAB_09010 (new NCBI locus tag CLSAB_RS04450; an identical sequence exists in strain NCP 258)

MDKSKEVYSEESLEENIDITFDDVVNFWMTLFPEVKSVEDFKKLLQNSSKTELSNLEKAYYKNSNADNTYKLFDNAFNRESNLEFMFDKIQKQYQWTYFFRPVIEYYSE
DLYEFIQKSLIIEDKEELFFGILSTTVKNLYELAFKVLILETNVARMENRLVGDTPKQKAGYFIKVLLRDKDYLMNLYSEYSELTRLMDLTVRNTFSYVKEIIESTGNE
MKSLEENFGQGKVIGKIANISLGEGDTHNRGKTVGVITFSSGIKLIYKPRLLDLENRFCEFVDWINKQKIPGFKELKACKLHTVNGAGWMECIENIECSTEEQVKNFYI
RTGQLLNILYMFNAKDFHYENLIAQGEYPILIDLETLLHPDMFNGNVEELSADAKAMKLISNSVKGIALLPTQIINNKTDKVLEVGGLCGESNQEAPFKSIFIKDHDTD
EICVEEGYGIIEAKMNNPIINGSKVASDLFIKEIKEGFENLYRWILRNKKVYADKIKELFEDCICRVIFRPTNVYCQLLSTSYHPDLLRNQTDRDVYLHRIGVIGTGKE
IIESELNDMFIGDVPYFSTYVNRNIVLNSRKEEIEFRYKKTTLDSILEKIEIVTEEDLDRQMALIDISFIDKSENKGRIFTKVNFQEEKVNLKLNKLQLLNTANLIGDY
IIEKSIVGGKDGVIDRTWIGSIEIGEKASFITPVGMDLYAGNSGIALFLAYLGSTTGEEKYKKAAIEAIEPIIRYLDELKDITNEKIGAFSGISGWLYSIFHIGHTLKN
QKLLDYVQKGISIMKELTGKTQCHDIISGYAGALGVMLSIYEKTENKELKMELISLCNTIFKELKDSIVVLKNQKGITWGEEGYVGYSHGNAGIEAQLMRLYSITKEDS
ILQIVKDSLSYERSMFDEKSNNWKKQLTKDEISYAWCHGAPGILLSRLMMVEAGYDDEKIRKEISIAMETTKRECFGMDYCLCHGDIGNLRILHYAANVLNDSTLKLQC
ETTLDVFINEYFIESFKQGNFKQTENVSLMLGPTGIGYGLLQFYKPDIMPEILRLG

Clostridium saccharobutylicum NCP 195

AQS16549.1; CLOSACC_44220 (new NCBI locus tag CLOSACC_RS21575; identical sequences exist in strains NCP 200, BAS/B3/SW/136, DSM 13864 = NCP 262)

MDKSKEVYSEESLEENIDITFDDVVNFWMTLFPEVKSVEDFKKLLQNSSKTELSNLEKAYYKNSNADNTYKLFDNAFNRESNLEFMFDKIQKQYQWTYFFRPVIEYYSE
DLYEFIQKSLIIEDKEELFFGILSTTVKNLYELAFKVLILETNVARMENRLVGDTPKQKAGYFIKVLLRDKDYLMNLYSEYSELTRLMDLTVRNTFSYVKEIIESTGNE
MKSLEENFGQGKVIGKIANISLGEGDTHNRGKTVGVITFSSGIKLIYKPRLLDLENRFCEFVDWINKQKIPGFKELKACKLHTVNGVGWMECIENIECSTEEQVKNFYI
RTGQLLNILYMFNAKDFHYENLIAQGEYPILIDLETLLHPDMFNGNVEELSADAKAMKLISNSVKGIALLPTQIINNKTDKVLEVGGLCGESNQEAPFKSIFIKDHDTD
EICVEEGYGIIEAKMNNPIINGSKVASDLFIKEIKEGFENLYRWILRNKKVYADKIKELFEDCICRVIFRPTNVYCQLLSTSYHPDLLRNQTDRDVYLHRIGVIGTGKE
IIESELNDMFIGDVPYFSTYVNRNIVLNSRKEEIEFRYKKTTLDSILEKIEIVTEEDLDRQMALIDISFIDKSENKGRIFTKVNFQEEKVNLKLNKLQLLNTANLIGDY
IIEKSIVGGKDGVIDRTWIGSIEIGEKASFITPVGMDLYAGNSGIALFLAYLGSTTGEEKYKKAAIEAIEPIIRYLDELKDITNEKIGAFSGISGWLYSIFHIGHTLKN
QKLLDYVQKGISIMKELTGKTQCHDIISGYAGALGVMLSIYEKTENKELKMELISLCNTIFKELKDSIVVLKNQKGITWGEEGYVGYSHGNAGIEAQLMRLYSITKEDS
ILQIVKDSLSYERSMFDEKSNNWKKQLTKDEISYAWCHGAPGILLSRLMMVEAGYDDEKIRKEISIAMETTKRECFGMDYCLCHGDIGNLRILHYAANVLNDSTLKLQC
ETTLDVFINEYFIESFKQGNFKQTENVSLMLGPTGIGYGLLQFYKPDIMPEILRLG

Clostridium sp. BL8

EQB90210.1; M918_01645 (new NCBI locus tag M918_RS28815)

MEITIEDIISFWTKIVPEASSKQELRELCIKASGEDIIDLANRVLKPDRDYKDILHILDEEKFYKLDEIVSEYRDTIPFFFFYEPYINTSLRKWFDSIYSLDILYDINM
FLKQCIYNLLRQLASMAARTLILEINIARMSEKLQGNTEKERFSYFHYKMLTNREYCSSLYFEYYELSKIMAQKTNDYFAYIYEMLTNIKSEVGIISRHYNIEKEKLII
ESISTSAGDEHISGKAVAIINFIENFKLVYKPRNLGFDIGFQNILEWIEIKSKGSVLPMKKLKIIERREFGLVEYVDNNECYSVEEIKEFYIKIGQYIAILHSLNAVDF
HSENIIADGKDPVLIDLETLFHPYVKIHSEEMYTESEKVANEVIENSVQSIGILPFYIMNEAVSDAKMDISGLGGAMEQKSPYKSYVVNNEYTDRIEVSREHLNIEPDK
NNPKLNGEIQKSENFVDSIIQGFKACYATILDNRMDYSRKVNNAFIGNTNRIIIRPTRDYTQLLNTSYHPDLLRNEEDRIVFFSRLGGNVSEERVKILRLELKSLLNGE
VPCFNCKIDSKHVYDVRNEVYVNFIEECPIDIVKKRIASFSDEDLVRQIKFIEVAFKTKESDYEKDKTFIDFNNVELGSSNISKEKYIELAVQMGDYILKDSILDKSET
NNRTWISAILKGRNESCLVLNPIGDDLYNGNAGVALFLIYLGYSTGQDKYVEAASQALESRRVFIDKAPKSYPFSIGAFNGLSGTIYTLDKLITYGKRSCDIEYIGKYV
RYLDDIIYMDKQYDLMGGALGCIAVLLPMIRRKAYPQFESIMKSVVTKCCKHLIKNGYKADVGGITWGDIMKSTGFSHGNAGVVAYLKQLANENWLQDVKNLDFVIDEA
LQFERALYVPEVRNWYKTNEKEEVAFGWCHGAPGILLSKCLLKSFGFHDNKLDDEIEHAIQTTKNNGFGNNPSLCHGDLGNLEILYIASKVLKDEKLQEECMHVFDDIF
EKVIAKRWNGQSYRGIESFSLMVGLAGFGYSLLKFYKPDSIPSILWLE

Clostridium sp. BL8

EQB90214.1; M918_01665 (new NCBI locus tag M918_RS28825)

MMEVDCIAKVKEILEHFNETQIEGILQTDKLDKTNIFDNFYKEYLWYFEINLKSILSDLKNLDFGMKEDKIVFSCLQSLRNDLIDISKKILISELYKMKEKDQLIGKNK
YERYEYFNSLLTGNKQLEIIEANPVMAFLIINKLQTKLNLIKECFERFACDYKEIKYAFDLDIKNLENIWLDEGDTHNDGKSVIILEFNYKKKLVYKPHTMAPDESFGK
LVNYINDSRYLKCPLKVAKAINKGRYGWQEFIKHKQCDNINQIKDHFYRIGVLLSIFNIIKSKDFHYENIIANGEYPVPIDLETILSNSKNELSVHEYGLAEAFVKEID
QSIYGSLMIPQNLEMFKFSVDLSGINGGSYEAQSIEFTRILNTGTDEIGYERVKDSIGEKQNRVKYNGKVVELQEYIPDIEKGLSDGYDFFIENKEKLISLIMDGKVFS
GEYRQVLRATANYVKFIDAAIHPVYTNDFESRIKVFNYLYGKGQLTDERKTRVNSEINQLLKNDVPYFWAKFDTHDLHSADGTCLKDYYQRTICESVVGKIQLADKKEK
EKQILYMKASIASLISIQNSNKYTEKYANDIFSNMVEHHNKSDRYIQIAKKIGEYLADMAIWNSDKDKCSFMALNYQTDGCIKYGPLNVKLYEGSGLLLFLSQLSRLTG
DNKYKKIVESAILGFDELFNKIELQITSEGVFTGIGSLTFTYYSLWTITKDKLFYDRYKECLKRLIDFDFSKSEVIDVIDGVAGLSIMGSNIYEKENDDMLLELMEKCG
EKLYSELAEEKDGYLTGFSHGYAGFSTALFMLAQHLNNEKYYNLGKDLVRKENEYYSEEKKNWKDLRPNHHEADPVFWCHGAAGIALSRAISKEYLKENDKCFLDRDID
LAVSKTLEYGFTSDMNQSLCHGSFGNIDCLLSVAIKTKDLELLERVYLTVDAECEKIIKNGIECANPLRVETINFMLGISGVGYQLLRLYDNNIPSILSLQV

Lysinibacillus sphaericus B1-CDA

WP_081010938.1; APF94_RS22610

MQQLNLNTTEDEKEILSFWKDFFPEFKEDDDLEKLFLRVYNNNIENYLKDREIEDTYQKEFINKIMAFNIKNDNEIIFNNIMDKYFHDFKWIGFFRPLLLKYDDTITSI
INSKLFKDSERAFDLIYCQMLQSLVNRSFRVIVLEINLARQENILLGKDSEERYTYFVKELLLDNNFLTKIYNKYPELIRILDNYLENTTKYLNEILKNFNDEKNSLFM
QMEINKDIKLESIEFSHGDTHNGNKSVAKLVFENNFNLMYKPRSLEIEKGYKDLIDWLDNRIDGFKKPYAANVYSNNNYGFMEFINNKECDNPEEISNFYFKMGELLAI
LYSLNSRDFHIENIIAFGENPVLVDLETLLHKTVEVDEEEISSYENILSIIGNSVASISVLPTTMINKKENLIMEVGAMNSGKVQKSPYRTQALEERNTDRIHIKNVYK

EIDKLNSSPKSGGRYLSAENYISEIKEGFIKIYRWIEENKDKYYKKIESLFADKYSRYIYRGTNNYTQLLETSYHPDLLSNKIDRYIYFHRIFTTIDFHNNEKDIGVTK
TEILEMLNDDIPMYQIRNDEIYISDMKGNIVLREEGRSILESIEERINSFNEIDLKRQISIINQCFIGCGLKTDIPDGTNTVFDESIQPRTRTENLKIAIDLTNSMLER
GIRNSSKESMSWIGLKGYGNELYENLPLEIDLYNGNSGIIITLIELYKISNEEKYKIAIVESVNYVLEFLETCNVEEVDLGAFSGLYGSFYSLFIAWKSNILPNNDRLI
KTLMKKLEESEKYIKQLNNLDIIGGLAGILGVLITLKDNLTNENPYIDQCLNRIIQTTYTRIEKSAIYVNENMVKWNENHDSGYAHGSSGIITQLVRYYKKYPDIRKLD
LIKKSLNYERAYLINESRNKWIIRDNSHYFSWCNGIGGILLTKLYLLNNNYSDEKIKAEIDILIEQLKLCGFRYDKSLCHGDLGSLIILKYAGEICGNTGLVNSSQNLI
SEVVYKYNDLKKDISMEIEDWGLMTGTSGVILSMLNIVGTIDITKIFLLEDPNIGEVR

Lysinibacillus sphaericus B1-CDA
WP_081010939.1; APF94_RS22615

MIINEKVLDQEIDFQDVLEYWSKFFPEVKTQIEVEDLIHQTTNRKFVDFKEEWEKESMRFALNKQKIDSLFNFKPNNKLFRILSTLKKEDFPFLNFFKPLFLREFGKWL
DHFEKFDLVYSKRTFYSDIFIHMYNISFNISYKMLVFEIDFEKKQGNLIGETPEDRFQYYINEKLQNEEYLKSLYMEYESLTNLLLLTINNYLKFLFDILSATTQEIKN
IFKTLFNTENIKKLINVSLNMGDTHNNGKTVSILTFEDNKKVVYKPRSLAFDQKYSDLLSWMENNQSKHLKKIKSAKIYTTSNHGWMEFIEYKPCNNIEQVKSFYIRSG
QLLCLLYALNSKDFHSENLIASGEYPYLIDLETLLQPDFEEKLSEKTVINKTQDFINRSVYSIYLLPSRMTINKNENTIKVVDFGGMAGYTKQQAPIKSNILINKNSDN
IRVIKDYGILDINDNNPFLNGELIQSELFIDDIQSGFTNMYRWITENKISFINKLCELFANEKCRVVCKPTFIYSQILKSSFHPDLLRNPIHREVYFSRIALSELKKGF
KHLSYHEHRDLMNGDIPYFSVYTDKKVLLDSKNSIIPNFEFKYSTLETVINKIQLMNELDLKKQITLIDLSYMHTSINDAKQETNLKFKTSKDFSNDAKPSKTDFLNTA
IKIGDRLLNESIYDTESTDRTWLGLMVYGKNEVTSHVSSIDIDLYKGNNGISLFFASLASITKLERFKKAAIETLSPSLKTLKNIEKNKEISLNIGAFTGLSGILYTIY
HVGNILEIEEYNNIALHYIDIVLEHLGDDTQIELLGGMSGALSVFISIYNTSDNAKVKKKLLDACYIIFEKILESTIEFKDNCVFWGYVDTNGDGGYTGFAHGTSGIIA
SLGRFYKITHDKNVYQLIKKALNFERELYSPTTKNWRTQINGEADSIGWCHGAAGILLSRSVLINHGYFDEQISKEINIALETTLKKGFGNNITLCHGDLGSIGVLFHL
ATVLDDETLYEQCNETFNEIFNHYISRKWDKTSYRTASVYGLMIGLAGYGYSLLKYGCRVKMPDILWIE

LanM homologs with established functions

Bacillus licheniformis DSM 13 = ATCC 14580 LanM2
AAU42941.1; BLi04128 (new NCBI locus tag BLI_RS20300)

MNEKSAGYHERLPVAQTQSPLVNDKIKYWRSLFGDDDKWLNKAVSLLSHDPLSSIAQSSVSQSVGLKDSRRGPWQKMQKRIFETPFSYKDSALQDSELLFDSLLTRFAS
AAQDALEEQNIILSPPLCRQVLTHLKQTLLQIAHQTLILELNILRLEDQLKGDTPEMRYLDFNDNFLVNPGYLRTLFNEYPVLLRLLCTKTDYWVQNFSELWKRLRQDR
EQLQAAFHIAGDPVHIELGVGDSHNKGKMAAILTYSDGKKIVYKPRSHDVDDAFQLLLSWINDRNSGSPLKTLRLINKKRYGWSEFIPHETCHTKKELEGYYTRLGKLL
AVLYSIDAVDFHHENIIASGEHPVLIDLESIFHQYKKRDEPGSTAVDKANYILSRSVRSTGILPFNLYFGRKNRDKVVDISGMGGQEAQESPFQALQIKGFFRDDIRLE
HDRFEIGEAKNLPTLDHQHVPVADYLHCIIEGFSAVYRLISDHGESYLATIEHFKNCTVRNILKPTAHYASLLNKSYHPDFLRDAVDREVFLCRVEKFEDADTDIAAAK
TELKELIRGDIPYFLSKPSDTYLLNGEEEPIAAYFETPSFTRVIKKISSFSDQDLKEQANVIRMSILAAYNARHEKDAIDIDQNHPSPRSGALQPLAIAEKAADDLAEK
RIEGNDGKDVTWISTVIEGVEEISWTISPVSLDLYNGNAGIGLFMSYLSRFAKRPETYSHITEQCVFAIQRALNELKEKEEFLKYADSGAFTGVSGYLYFLQHAGTVQK
KNEWIELIHEALPVLEAVIEQDENCDIISGSAGALMVLMSLYEQLDDPVFLKLAEKCAGHLLQHKTNIENGAAWKDPHTQNYYTGFAHGTSGIAAALSRFNKVFDSQSL
KKIISQCLAFEKQLYIASEKNWGSKGREQLSVAWCHGAAGILLSRSILRENGVNDPGLHTDILNALETTVKHGLGNNRSFCHGDFGQLEILRGFREEFSELNTIIQNTE
DRLLTYFQENPFSKGVSRGVDSAGLMLGLSGVGYGMLQCQYGEELPELLQLSPPQALIKKNSKAFKRENVF

Lactococcus lactis subsp. *lactis* C2102
AAC72258.1; LctM

MKKKTYQFEKFLKNTFDQFSIKQNEVLVEDDLNDIIMNVCGKALVLMINEKREMNLLMGNTPEERYQYFENEYSSTGKAFEEIKDKFPVIYIDLKNSINSYLKLVSQIM
KDFKKDYSLLVERKIIEEHSTISTMKIKGDLHNGKAVIEITTNKSKLIYKPKSLSNDVFFNNFLKYMDSFFIKEGKSTKYKENFYLVNTLDMKTYGWVEYVDKKPINSF
EEARNYYRKIGVLLSVAYTLNLTDLHFENVISQGENPCIIDLETMFNMPMFVKDYKNESRNIINGKIMDSVVSTGMLPVLGIDSLFGGDPSGILGGTFSKEERVIINPF
RDDIKFQKIVVRSVFKDHIPFFNNNNEKRYCKPKDYVNDIIKGFEKTYKIIVKNKEKILGFLKKESSSVTCRILFRNTMEYSVLLNAAKSPVYSNKREEIFEKLSTFNR
GLGNDIIKSEISQINTLSIPYFNCQVDSNLIKNMDGETIFEHTLTPFKCFLSKYRRLCVDDMEQQVKLIRFSIQSQEQLFKDGEQFSLYKKQKGSQEDLLIAINELSSI

LENNAYIGTSDDTINWMSLGIADNDQILFESLENDIYKGISGIGLALLEYYEFYPNINTKKILKLIYKNISKDFINTNNEPQNYGFYVGLIGEYSFLRKYEKVFHKTSS
CNILKNILKDFTPEKCQTILPSDDVIAGEAGIIIYISNLNNYLEYRDEIDILLKSLSNKIKLKESIASYAHGNSGIATAFVHGYKVTKNEKYLKIFHELWNLENSSKLR
RGWTDSRKVDSSYSSQWCHGASGQAIARMEWITVNKTARFLSNSELIKVKKELGELIDILKKEGMYTDNFCLCHGILGNLLILNTYQENFDNKNINLKNEILNNYYSVC
NYGLNKGWICGLGTEFYSYGLMTGISGILYGLIRQVKQKNNFGVLMPYVD

Bacillus licheniformis DSM 13 = ATCC 14580 LanM1
AAU25565.2; BL00928

MSMKEFEIYLYKALYSNERGGQGQEHPSGFFPENGKTPSRPTDFHLSSVQHSPNEPVQLQGKMPEWAACLSEIMKYNPKAVSELKHPLPHMSFVTFFVPFLLFAQERMS
KAFSEFEKQEGGLSGIIDAAGYQDGIMSELHQCLDKLATRTLITELNVAREDGRLKGASPEERYVYFVEQYISDPEIYREFFELYPVLGRLMAEKVLRVLEIHEEIIGR
FLSDRSLIAKKFNIASPELVGFEGDLGDSHKNGQSVKVLVLNNGKLVYKPRSLSIDEHYRELLNWLNGRGMKYSLRAAEVLDRGNYGWQEFVKHEGCSSEEELERFYFR
QGGHLAILYGLRSVDFHNENIIASGEHPILIDLETLFDNHVSIFAQNQNLHVTALELKHSVLSSMMLPVKFKHDEVLDFDLSGIGGKGGQQSKKAKGYAVLNYGEDRMS
LKETSLTTEEKLNAPKLNGRPVSAVFYTDFIVEGFKNAYAIMMKHKEELAGPSGFLNLFKHDEVRHVFRPTHVYGKFLEASTHPDYLTAGDKREQLFDYMWMLAKQSEK
ANVFIPDEIVDLLLHDIPYFTFYAGGTSLLNSRGEESEGFYETSSIDLAKKKIQSFSEKDLNHQLRYISLSMATLIENVWDHAESGLGQKETVADLGKEVKHIADDLLQ
KAIYSERGEGPFWISNNAGDEKMVFLSPLPMGLYDGMAGLAIFFAQAGKVLNEQVYTDTARSMIEEIQKEESYWVQNGNSHSAFFGTGSFIYLYSYLGSLWEDDSLLER
ALNLIPRVLDQPNQTQNPDFIAGDSGLLTVLVNLYEIKQHPAVLDSIRQVLSRLNDRIGRLLDSIEQDAVSLTGFSHGLTGIAFSIAKAAKVIHDDSCKELVLKLVEEE
DRYFQKDHLNWLDLRNDSHTLSPSYWCHGAPGILLGRAHIQAFIPELTTRTLKLQEALQSSLNLADCQNHSLCHGLIGNLNILLDIKRLNRELHVPDDIFCIYKTKNRG
WKTGLHSDVESLGMFVGTAGIAYGLLRLLDESVPSVLTLDIPTGR

Table S2. List of AgrD homologs used for Figure S1.

Clostridium acetobutylicum ATCC 824
AAK78064.1; CA_C0079 (identical sequences exist in strains ATCC 55025. DSM1731, DSM1732, EA2018, GXAS18-1, JB200, NCCB24020, WA and others)
MNLKEQLNKVNDKFIKGLGKASMKIGEQANGKCVLVTLYEPKMPEELLKENIDK

Clostridium felsineum DSM 794
OOL89487.1; CLFE_29770 (alternative accession and locus tags numbers are URZ14059.1 and CLFE_000340)
MNLKKQVNKIGEKFIEGIGKASMKIGEQASDTCVLITLYEPKMPEELLKENLNK

Clostridium aurantibutyricum DSM 793
OOL97528.1; CLAUR_41060 (reclassification as *C. felsineum* DSM 793 proposed; alternative accession and locus tags numbers are URZ02707.1 and CLAUR_027310)
MNLKKQVNKISEKLIEGIGNVSIKVGEQATDICVLATLYEPKMPEELLKENIDK

Clostridium roseum
OOL86543.1; CROST_26800 (reclassification as *C. felsineum* DSM 7320 proposed; alternative accession and locus tags numbers are URZ08971.1 and CLROS_043750; an identical sequence exists in strain DSM 6424)
MNLKKQVNKISEKLIEGIGNVSIKVGEQATDICVLTTLYEPKMPEELLKGNIDK

Clostridium sp. CT7
PJI08303.1; CUB90_10700
MSLKNQLNKVNDKVINSIGKASMKVGNRAFGVCMVFAIYEPKIPDALIKENYKRK

Clostridium beijerinckii NRRL B-593

OOM53264.1; CLOBI_50580 (identical or >94% identical sequences exist in strains NRRL B-528, DSM 6423, CloBei18h, DJ028, DJ030, DJ031, DJ034, DJ035, DJ051, DJ124 and DJ323)

MKSMRKQITSTGERLSDKIINGICDVSIKIGEKSRGRCSMLGAYEPKISIDLLKEENK

Clostridium diolis NJP7

WP_087702269.1; CCS79_RS13975

MKSMRKQITSTGERLSDKIINGICDVSIKIGEKSRGRCSMLGAYEPKISIDLLKEENK

Clostridium cellulovorans 743B

ADL53915.1; Clocel_4254

MRNMREELIKIGDMVIGEIGNEAVKLGDRARGLCLFLNVYEPVIPIELLDDIE

Clostridium hungatei DSM 14427

OPX45184.1, CLHUN_10710

MKNIIGQFASMREKVEDKLISSIGNTATRIGEQSRGLCLLVFDYEPKFPMELLMKNDEQ

Pseudobacteroides cellulosolvens ATCC 35603 = DSM 2933

KNY28928.1; Bccel_4202

MRNMKEQIVSMREKIGSKILGEIGNATVKIGEQSRGFCILLFSYEPKVPKELLYEKIED

Table S3. Optical density of wild type and CA_C0082, *agrC* and *glg* mutants strains after 24 hours of growth at different glucose concentrations.

Strain	OD_{600} after 24 h of growth ± SEM<br>Glucose concentration [w/v]				
	1%	2%	3%	4%	5%
C. acetobutylicum ATCC 824	2.25 ± 0.06	2.33 ± 0.07	2.39 ± 0.06	2.63 ± 0.12	2.46 ± 0.11
C. acetobutylicum CA_C0082::CT*ermB*_s	2.21 ± 0.03	2.11 ± 0.07	2.47 ± 0.12	2.44 ± 0.16	2.50 ± 0.08
C. acetobutylicum *agrC*::CT*ermB*	2.11 ± 0.09	2.39 ± 0.12	2.38 ± 0.15	2.64 ± 0.17	2.81 ± 0.09
C. acetobutylicum Δ*glgA*	2.40 ± 0.08	2.35 ± 0.04	2.41 ± 0.09	2.46 ± 0.12	2.36 ± 0.07

Table S4. List of Oligonucleotides.

Oligonucleotide	Sequence (5'-3')
	Verification of ClosTron mutants
Ca_c0082-sF1	AAGTGGTAATAGTAGACG
Ca_c0082-sR1	CTTCTATCCCATCTAGCA
EBS universal	CGAAATTAGAAACTTGCGTTCAGTAAAC
	Construction of complementation vectors
Ca_c0082-F1	GTTTCTTCGAATTCGCGGCCGCACTAGACATTCTGTAAATAGGCAAATAGAA
Ca_c0082-R1	GTTTCTTCCTGCAGCGGCCGCGCTAGCTTATTCCAACCATAAAATTTTCGGAAC
Ca_c0082-F2	AATTTTTAAGGAGGTGTGTTACATATGTTGGGAAGGAGAAAAAATG
Ca_c0082-R2	GCAGGCTTCTTATTTTTATGTTATTCCAACCATAAAATTTTCG
	Construction of gusA promoter fusions
P_Cac0082-F	AAAAGCGGCCGCAAACAAGCATTACATTACATAAAATCCAACG

P_Cac0082-R	AAAAGAATTCATGAATATATCATAAATATGTGATAGCTAATTATT
